# MdaB and NfrA, Two Novel Reductases Important in the Survival and Persistence of the Major Enteropathogen Campylobacter jejuni

**DOI:** 10.1128/JB.00421-21

**Published:** 2022-01-18

**Authors:** Fauzy Nasher, Aidan J. Taylor, Abdi Elmi, Burhan Lehri, Umer Z. Ijaz, Dave Baker, Richard Goram, Steven Lynham, Dipali Singh, Richard Stabler, David J. Kelly, Ozan Gundogdu, Brendan W. Wren

**Affiliations:** a Faculty of Infectious and Tropical Diseases, London School of Hygiene and Tropical Medicine, London, United Kingdom; b Department of Molecular Biology and Biotechnology, University of Sheffield, Sheffield, United Kingdom; c School of Engineering, University of Glasgow, Glasgow, United Kingdom; d Microbes in Food Chain, Quadram Institute Biosciences, Norwich, United Kingdom; e The John Innes Centre, Norwich, United Kingdom; f Proteomics Facility, Centre of Excellence for Mass Spectrometry, King’s College London, London, United Kingdom; University of Chicago

**Keywords:** *Campylobacter jejuni*, flavins, MarR-like regulators, MdaB, NfrA, quinones, RrpA, RrpB

## Abstract

The paralogues RrpA and RrpB, which are members of the MarR family of DNA binding proteins, are important for the survival of the global bacterial foodborne pathogen Campylobacter jejuni under redox stress. We report that RrpA is a positive regulator of *mdaB*, encoding a flavin-dependent quinone reductase that contributes to the protection from redox stress mediated by structurally diverse quinones, while RrpB negatively regulates the expression of *cj1555c* (renamed *nfrA* for NADPH-flavin reductase A), encoding a flavin reductase. NfrA reduces riboflavin at a greater rate than its derivatives, suggesting that exogenous free flavins are the natural substrate. MdaB and NfrA both prefer NADPH as an electron donor. Cysteine substitution and posttranslational modification analyses indicated that RrpA and RrpB employ a cysteine-based redox switch. Complete genome sequence analyses revealed that *mdaB* is frequently found in Campylobacter and related *Helicobacter* spp., while *nfrA* is predominant in C. jejuni strains. Quinones and flavins are redox cycling agents secreted by a wide range of cell types that can form damaging superoxide by one-electron reactions. We propose a model for stress adaptation where MdaB and NfrA facilitate a two-electron reduction mechanism to the less toxic hydroquinones, thus aiding survival and persistence of this major pathogen.

**IMPORTANCE** Changes in cellular redox potential result in alteration in the oxidation state of intracellular metabolites and enzymes; consequently, cells make adjustments that favor growth and survival. The work we present here answers some of the many questions that have remained elusive over the years of investigation into the enigmatic microaerophile bacterium Campylobacter jejuni. We employed molecular approaches to understand the regulation mechanisms and functional analyses to reveal the roles of two novel quinone and flavin reductases; both serve as major pools of cellular redox-active molecules. This work extends our knowledge on bacterial redox sensing mechanisms and the significance of hemostasis.

## INTRODUCTION

Campylobacter jejuni is a microaerophilic Gram-negative bacterium and is the leading cause of bacterial foodborne gastroenteritis worldwide (1). The high prevalence of C. jejuni is credited to its ability to survive in a variety of niches, including the natural environment and within its avian and mammalian hosts, despite not growing in aerobic environments (2).

The sensitivity of C. jejuni to both oxygen and oxidative stress is a major defining feature that has presented a conundrum in terms of the prevalence of the bacterium in the natural environment and its success as a global enteric pathogen. Genomic data, mutant phenotypes, and biochemical analyses have shown that C. jejuni strains have an extensive complement of oxidative stress protection systems (3), which allows fine-tuning of its adaptation to *in vivo* and *ex vivo* environments. These include the peroxidatic enzyme catalase and the thiol peroxidases Tpx, Bcp, and AhpC, as well as superoxide dismutase (4, 5).

The production of most of the above-mentioned oxidative stress protection enzymes is controlled at the transcriptional level by the regulators PerR, CosR, and Fur (3). However, the reannotation of the C. jejuni NCTC 11168 genome led to the discovery of two novel redox-sensing MarR-homologue transcriptional regulators that were named RrpA (*cj1546*) and RrpB (*cj1556*) (6, 7). Using C. jejuni 11168H, a hypermotile derivative of the standard strain NCTC 11168 (8, 9), a link between RrpA and RrpB with oxidative stress was reported based on decreased viability in mutant strains after treatment with oxidative stress-inducing compounds, hydrogen peroxide, menadione, and cumene hydroperoxide (6, 7, 10). Additionally, multilocus sequencing typing (MLST) revealed that *rrpA* was present in over 99% of 3,746 C. jejuni strains, but the presence of *rrpB* predominated in livestock-associated strains (10). This suggests a correlation between possession of both transcriptional regulators and the ability of C. jejuni to adapt and survive in diverse niches.

Here, we asked what genes are under the regulatory control of RrpA and RrpB and what their roles are in C. jejuni redox metabolism. We identified *mdaB* and *nfrA*, encoding two novel reductases that are under the transcriptional control of RrpA and RrpB, respectively, and we revealed the regulation mechanism of RrpA and RrpB to their DNA substrates. We show that MdaB is a flavin-dependent NADPH-quinone reductase which has a role in protecting the cell from quinone stress-mediated damage, while NfrA is an NADPH-riboflavin reductase. Database searches revealed that MdaB is predominant in both Campylobacter spp. and the related *Helicobacter* spp., but NfrA is more often found in C. jejuni strains commonly associated with human infection.

Quinones are among the main chemical compounds produced by plants and microorganisms and have attracted significant interest because of their antimicrobial activities (11). Quinones are well known for their active role in the electron transport chain of most organisms in the form lipid-soluble electron carriers (e.g., ubiquinone and menaquinones) (12). In contrast, water-soluble quinones are toxic as prooxidants or electrophiles (13–16) and act as catalysts to generate reactive oxygen species (ROS) by undergoing one‐electron reduction to yield semiquinone radical anions that reduce molecular oxygen. This produces ROS such as the superoxide anion (O_2_^·−^) and hydrogen peroxide (H_2_O_2_) that lead to redox cycling reactions (17, 18).

Flavin mononucleotide (FMN) and flavin dinucleotide (FAD), and rarely riboflavin itself, form an integral part of the redox active sites of flavoproteins as prosthetic groups (19). The core of flavin compounds consists of a heterocyclic isoalloxazine ring that exist in three redox states: oxidized form, one-electron reduced radical semiquinone, and two-electron fully reduced hydroquinone (20). In fact, this property makes flavin molecules unique compounds in nature and fit to serve in broad roles as biocatalysts. However, like quinones, flavo-semiquinones can transfer an electron to oxygen, generating the superoxide radical (16, 21). Additionally, free flavins have been shown to transfer electrons to convert Fe^3+^ into Fe^2+^ and O_2_ into H_2_O_2._ The simultaneous production of H_2_O_2_ and Fe^2+^ in cells may promote the production of hydroxyl radical via the Fenton reaction, resulting in cell death (22).

Given their propensity for highly toxic superoxide production, both quinones and flavins are effective antimicrobials (23, 24) that C. jejuni may encounter in *in vivo* and *ex vivo* environments. Thus, we propose that possession of the MdaB and NfrA enzymes allows a safer two-electron reduction to the fully reduced forms of these compounds and could be a significant adaptation for the persistence and prevalence of this problematic pathogen.

## RESULTS

### Disruption of *rrpA*, *rrpB*, and *rrpAB* shows altered transcription profiles in C. jejuni 11168H.

The pleotropic phenotype of the C. jejuni Δ*rrpA*, Δ*rrpB*, and Δ*rrpAB* strains (6, 7, 10) led us to examine the transcription profiles of the 11168H wild type and its respective Δ*rrpA*, Δ*rrpB*, and Δ*rrpAB* defined mutants by transcriptome sequencing (RNA-Seq). Bacteria were cultured in brucella broth at 37°C under microaerobic conditions to an optical density at 600 nm (OD_600_) of ∼0.45 (mid-log growth phase), and five biological replicates were analyzed. Genes that were significantly differentially regulated between the wild-type and mutant strains are presented in Table 1 (genes that were differentially expressed >1.5 log^2^ fold change compared to the wild type were considered significant; *P < *0.05).

**TABLE 1:** Significantly differentially expressed genes at the mid-log growth phase in the mutant strains Δ*rrpA*, Δ*rrpB*, and Δ*rrpAB*^*a*^

Strain	Gene name	Product	Function	Log^2^ fold change
11168H *ΔrrpA*	*cj1677* (*capA*)	CapA—Campylobacter adhesion protein	Adhesion protein	2.17
	*cj1545c* (*mdaB*)	MdaB—NAD(P)H-quinone reductase	Putative reductase	–1.98^*b*^
11168H *ΔrrpB*	*cj1556* (*rrpB*)	RrpB—MarR-like transcriptional regulator	Putative transcriptional regulator	3.85
	*cj1555c* (*nfrA*)	NfrA—putative NAD(P)-dependent reductase	Putative reductase	2.81
	*cj1677* (*capA*)	CapA	Campylobacter adhesion protein A	1.88
11168H *ΔrrpAB*	*cj1556* (*rrpB*)	RrpB	Putative transcriptional regulator	3.17
	*cj1555c* (*nfrA*)	NfrA—putative NAD(P)-dependent reductase	Putative reductase	2.50
	*cj1719c* (*leuA*)	LeuA—2-isopropylmalate synthase	Amino acid biosynthesis	1.98
	*cj1546* (*rrpA*)	RrpA—MarR-like transcriptional regulator	Putative transcriptional regulator	1.77
	*cj1454c* (*rimO*)	RimO—methylthiotransferase	Ribosomal protein methylthiotransferase	1.69
	*cj1710c* (*rnJ*)	Rnj—ribonuclease J	An RNase that has 5′–3′ exonuclease and possibly endonuclease activity	1.69
	*cj1711c* (*ksgA*)	KsgA/RsmA—methyltransferase	Ribosomal RNA small subunit methyltransferase A	1.60
	*cj0724*	Putative molybdenum cofactor biosynthesis protein	Uncharacterized	–2.01
	*cj0265c* (*torB*)	Cytochrome *c*-type heme-binding periplasmic protein	Putative cytochrome *c*-type heme-binding periplasmic protein	–1.50

a For the complete data set for 11168H and the Δ*rrpA*, Δ*rrpB*, and Δ*rrpAB* mutants, see Table S1a to c.

b An individual statistical test indicated that the gene was upregulated (*P *< 0.05).

There was a modest difference in gene expression between the wild-type strain and its Δ*rrpA* and Δ*rrpB* mutants; the putative autotransporter gene *capA* (*cj1677*) was significantly upregulated in both the Δ*rrpA* and Δ*rrpB* mutants (Table 1). RrpB was previously reported to be an autoregulator (6), and interestingly, in both Δ*rrpB* and the Δ*rrpAB* mutant strains, *rrpB* and the gene directly upstream on the reverse strand, *cj1555c*, were significantly upregulated (Table 1). *cj1555c* codes a hypothetical protein of unknown function; however, a protein BLAST search (https://blast.ncbi.nlm.nih.gov/) indicated that this gene is an NAD(P)H-flavin reductase, which we have named *nfrA* [NAD(P)H-flavin reductase A].

Additionally, in the Δ*rrpA* mutant, *cj1545c* (*mdaB*; modulator of drug activity B) was downregulated based on an individual statistical test (*P* < 0.05); however, when the *P* values were adjusted for multiple comparison, the significance was lost. Nevertheless, this gene was included in the study (indicated with an asterisk in Table 1) for two reasons: (i) *mdaB* was previously implicated to have a role in oxidative stress defense in C. jejuni and in the closely related *Helicobacter* spp. (25, 26), and (ii) *mdaB* is located directly upstream of *rrpA* on the reverse strand in a divergent orientation.

In the double mutant strain, Δ*rrpAB*, genes that were significantly upregulated included *rrpA*, which was previously also reported to be an autoregulator (7), *cj1719c*, which encodes LeuA (2-isopropylmalate synthase), an amino acid biosynthesis protein, *cj1454c*, encoding RimO, a ribosomal methylthiotransferase that catalyzes the methylthiolation of aspartic acid residue of ribosomal protein S12, *cj1710c*, encoding Rnj, involved in the maturation and/or decay of mRNA, and *cj1711c*, encoding RsmA, which plays a role in the biogenesis of ribosomes and has been shown to protect DNA against oxidative stress in some bacteria (27, 28). Two genes were significantly downregulated, *cj0724*, which is an uncharacterized molybdenum cofactor biosynthesis protein, and *cj0265c*, the TorB cytochrome *c-*type heme-binding subunit of the TorAB, trimethylamine-N-oxide/dimethyl sulfoxide (TMAO/DMSO) reductase (Table 1). Lists of all genes are presented in Tables S1a to c in the supplemental material.

**FIG 1:**
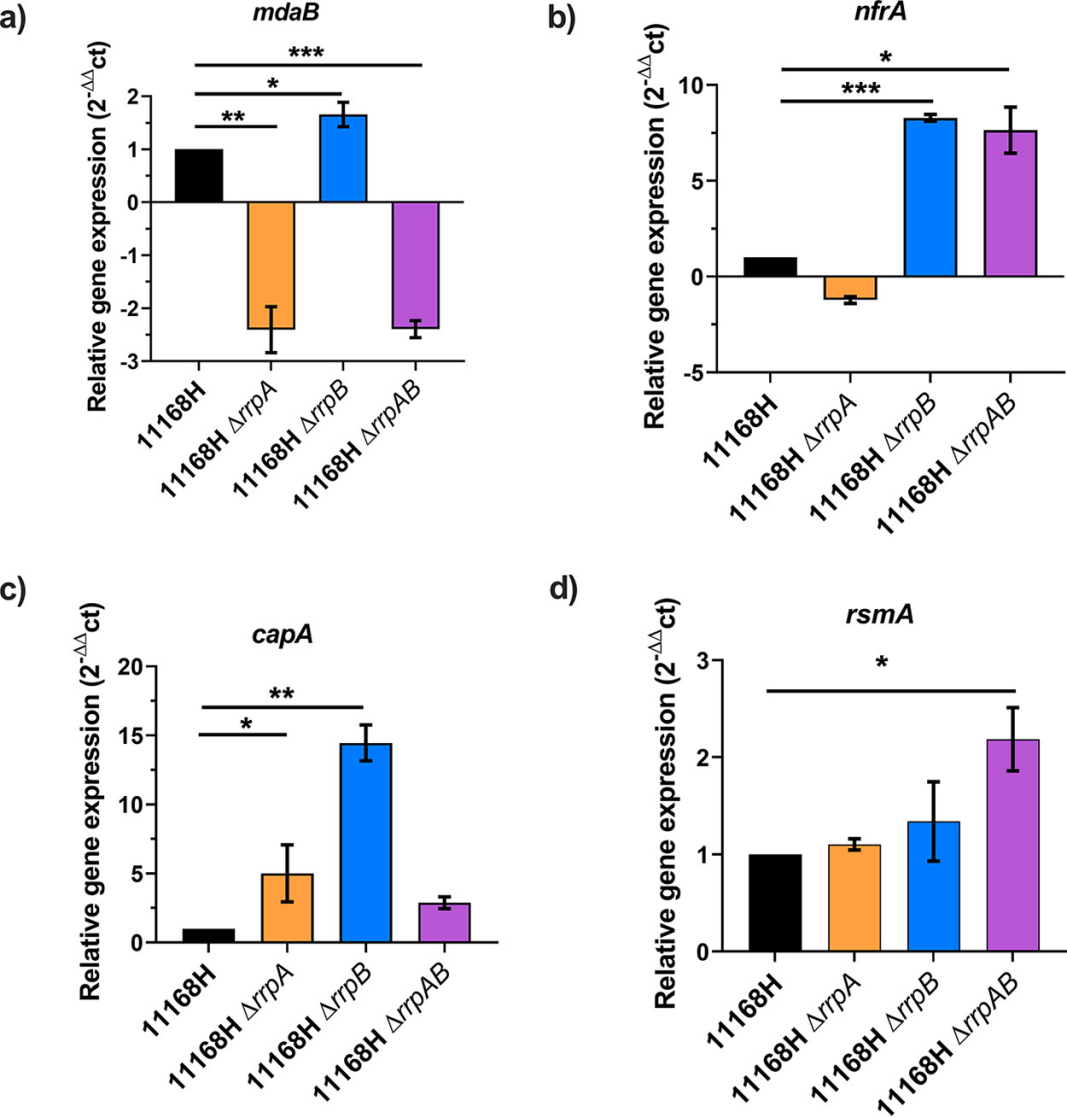
Relative gene expression of *mdaB* (a), *nfrA* (b), *capA* (c), and *rsmA* (d). Expression of the genes was determined by real-time RT-PCR and is displayed relative to the wild-type expression, after normalization with *gyrA*. The data presented are the mean value of at least 3 independent experiments performed on different days; error bars indicate standard deviation (SD). *, *P* ≤ 0.05; paired Student’s *t* test was used; **, *P* ≤ 0.01; ***, *P* ≤ 0.001.

RNA independent from the RNA-Seq was isolated, and real-time reverse transcriptase quantitative PCR (RT-qPCR) was performed (Fig. 1). RT-qPCR indicated a significant ∼2-fold decrease in the expression of *mdaB* in both Δ*rrpA* and Δ*rrpAB* mutant strains, while in the Δ*rrpB* mutant strain, *mdaB* was significantly upregulated by ∼1.5-fold (Fig. 1a). In both Δ*rrpB* and Δ*rrpAB* mutant strains, *nfrA* was significantly upregulated by ∼7-fold (Fig. 1b). Expression of *capA* and *rsmA* was also confirmed in all the strains, and their expressions were in line with our RNA-Seq data (Fig. 1c and d).

Our RNA-Seq and RT-qPCR results showed that the genes located upstream of *rrpA* and *rrpB*, respectively, were differentially regulated in the mutant strains. Therefore, we speculated that these genes are under the control of RrpA and RrpB. DNase I footprinting using dye primer sequencing on an automated capillary DNA analysis system was used to test the interactions of recombinant RrpA_his6_ and RrpB_his6_ proteins with the regions upstream of the genes *mdaB* and *nfrA*.

### RrpA and RrpB protect regions with inverted repeat sequences.

**FIG 2:**
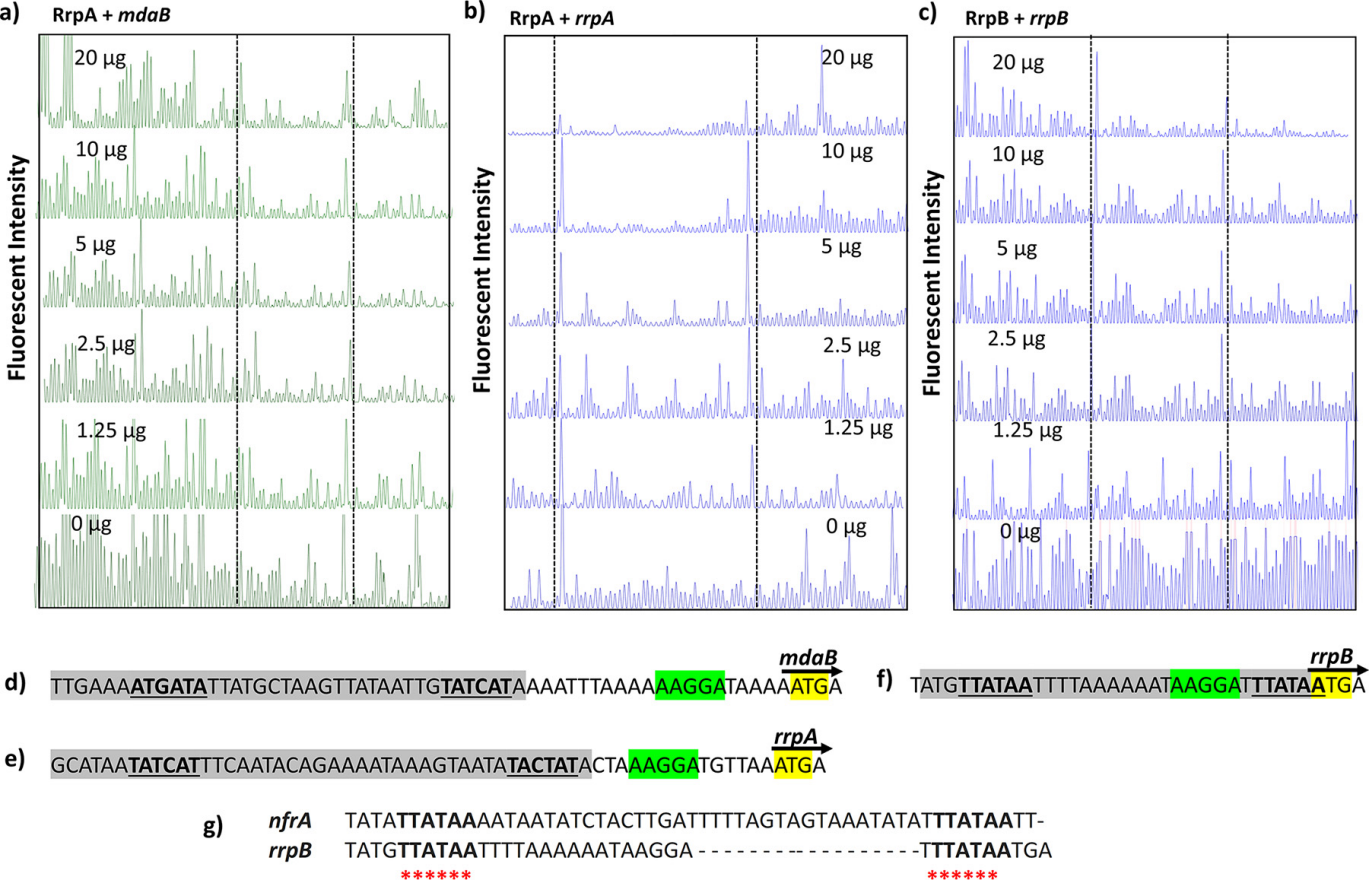
Electropherograms of fluorescent dye-labeled DNA fragments. (a and b) Reduced electropherogram signals indicate protection regions by RrpA_his6_ upstream of *mdaB* (a) and *rrpA* (b). (c) Protection regions by RrpB_his6_ upstream of *rrpB*. (Regions between the lines indicate protected regions; blue and green electropherograms indicate FAM and Hex fluorescently labeled DNA fragments, respectively. The electropherograms are presented as arbitrary scale). (d and e) The protected regions by RrpA_his6_ (gray highlight) including the inverted repeat sequence (bold and underlined) upstream of *rrpA* and *mdaB* transcription start sites. (f) The protected region by RrpB_his6_ (gray highlight) upstream of *rrpB* (inverted repeat sequences are bold and underlined). (g) The sequence alignment of the *rrpB* protected region and the region upstream of *nfrA* are matched (asterisks). Nucleotides highlighted in green indicate the C. jejuni ribosomal biding site (70), and yellow highlights indicate the gene translation start site. Electropherograms are representative of three experiments.

DNase 1 footprinting was performed as described previously (29); a 500-ng fluorescently labeled DNA fragment was incubated with various concentrations of recombinant RrpA_his6_ and RrpB_his6_ ranging from 20 μg to 0 μg at room temperature, as described in Materials and Methods. The pattern of protection was observed by decreased fluorescent intensity of the electropherogram (indicated between the dotted lines) (Fig. 2). We also noted nonspecific protection patterns at the highest protein concentrations in all of the samples, possibly due to promiscuity at high protein concentrations due to low binding affinity.

We observed protection by purified RrpA_his6_ upstream of *mdaB* and upstream of the *rrpA* translational start sites (Fig. 2a and b, respectively). Purified RrpB_his6_ showed protection upstream of the *rrpB* translational start site (Fig. 2c). In these protected regions (highlighted in gray), we identified an inverted repeat (IR) motif (underlined in bold) protected by RrpA_his6_ formed by 6 nucleotides (nt) (5′-TATCAT-3′), which are separated by 19 nt for *mdaB* (Fig. 2d) and 24 nt for *rrpA* (Fig. 2e); we also identified an IR sequence protected by RrpB_his6_ formed of 6 nt (5′-TTATAA-3′) separated by 17 nt (Fig. 2f). We did not observe protection by RrpB_his6_ upstream of *nfrA*, possibly due to oxidation of the protein during sample preparation. However, nucleotide alignment of the region preceding the translation start site of *nfrA* identified an IR sequence that matched those found within the protected region by RrpB_his6_, upstream of the translation site of *nfrA* (Fig. 2g).

**FIG 3:**
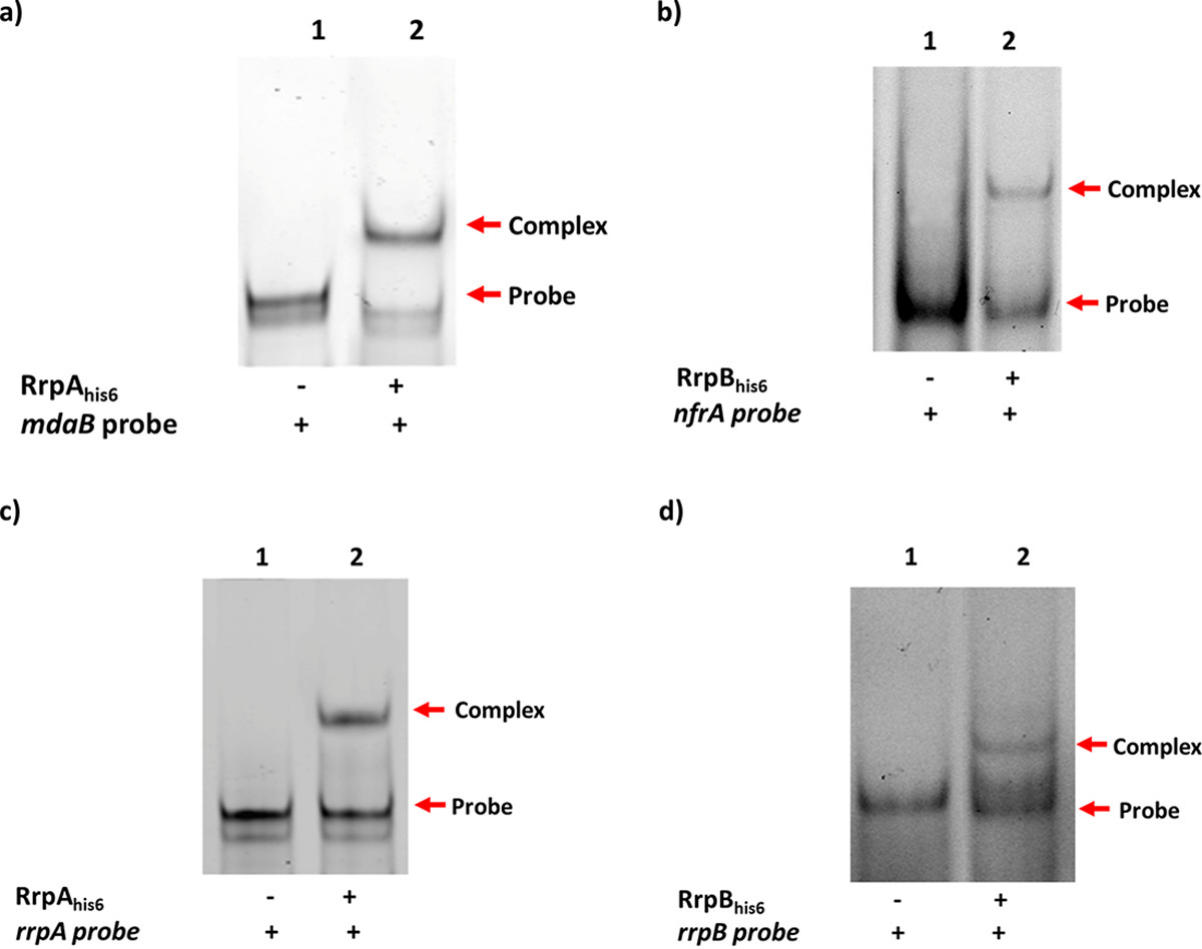
Electrophoretic mobility shift assay. The EMSA was conducted to confirm RrpA_his6_ and RrpB_his6_ DNA binding activity. (a and b) RrpA_his6_ (0.05 μg) binds upstream of *mdaB* (a), and RrpB_his6_ (0.05 μg) binds upstream of *nfrA* (b). (c and d) RrpA_his6_ binds upstream of *rrpA* (c), and RrpB_his6_ binds upstream of *rrpB* (d). Binding is indicated by a shift of band (arrows). IRDye 800 DNA fragments were used and imaged with LI-COR Odyssey imaging scanner (LI-COR Biosciences).

Oligonucleotides spanning the protected regions were synthesized, and electrophoretic mobility shift assays (EMSA) were conducted. A DNA substrate, 50 nM, was coincubated with a final concentration of 0.05 μg of RrpAhis_6_ and RrpB_his6_ proteins in a 20-μl reaction; formation of protein-DNA complexes was observed in all the EMSAs conducted (Fig. 3). Specificity of binding was also tested; we found that both RrpA_his6_ and RrpB_his6_ proteins are able to bind to DNA sequences with mutation to one of the binding sequences but were unable to bind to DNA that lacked both binding sequences (Fig. S1a and b). This indicates that RrpA and RrpB recognize regions on the DNA with IR sequences, which is characteristic of the MarR family transcriptional regulators (30).

### *mdaB*-*rrpA* and *nfrA*-*rrpB* gene expression respond to a range of exogenous quinones and flavins, respectively.

**FIG 4:**
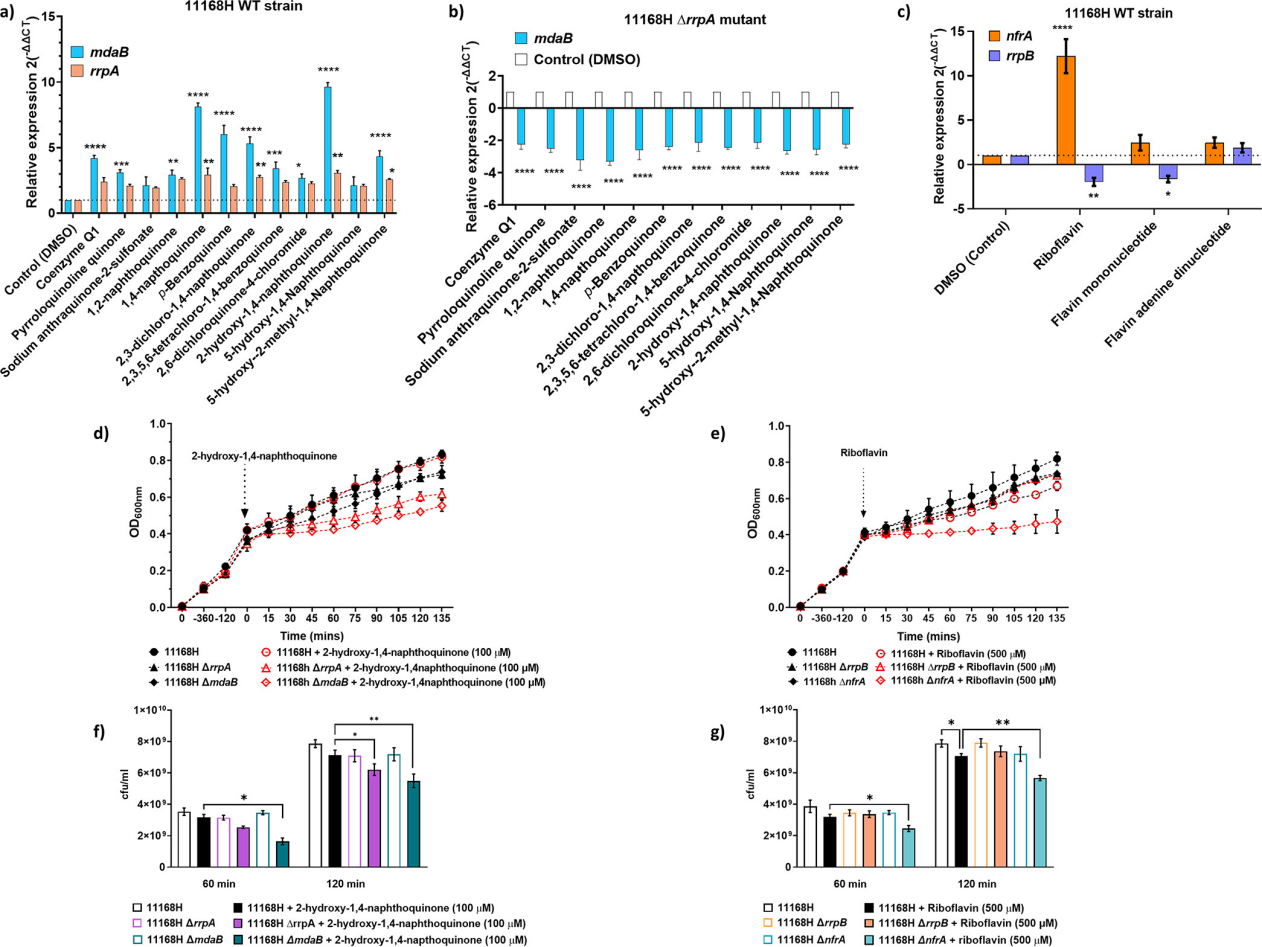
*mdaB* and *nfrA* respond to structurally diverse quinones and flavins. (a to c) Expression levels of *mdaB* and *rrpA* (a), *mdaB* in the Δ*rrpA* mutant strain 15 min after treatment with 100 μM quinones (b), and *nfrA* and *rrpB* 15 min after treatment with 100 μM flavins (c). (d and e) Growth in brucella broth was determined by measuring the OD_600_ every 15 min after addition of 100 μM 2-hydroxy-1,4-naphthoquinone to the 11168H, Δ*rrpA*, and Δ*mdaB* strains (d) and 500 μM riboflavin to the 11168H, Δ*rrpB*, and Δ*nfrA* strains at an OD_600_ of ∼0.45 (e). (f and g) Cell viability was determined 60 min and 120 min after treatment with 100 μM 2-hydroxy-1,4-naphthoquinone (f) and 500 μM riboflavin (g). Gene expression was determined by real-time RT-qPCR after treatment with compounds and is displayed relative to the value of the control expression, after normalization with *gyrA* expression. The values are the means of at least three independent experiments. Error bars = SD. *, *P *≤ 0.05; **, *P* ≤ 0.01; ***, *P* ≤ 0.001.

We performed a literature search to identify a broad range of quinones that have previously been shown to have an effect on *mdaB* analogs (25, 31). We selected the following compounds due to their structural diversity: coenzyme Q1 (ubiquinone), pyrroloquinoline quinone (methoxatin), sodium anthraquinone-2-sulfonate, 1,2-naphthoquinone (ortho-naphthoquinone), 1,4-napthoquinone, p-benzoquinone, 2,3-dichloro-1,4-napthoquinone (dichlone), 2,3,5,6-tetrachloro-1,4-benzoquinone (chloranil), 2,6-dichloroquinone-4-chloromide (Gibb’s reagent), 2-hydroxy-1,4-naphthoquinone (Lawsone), 5-hydroxy-1,4-naphthoquinone (juglone), and 5-hydroxy-2-methyl-1,4-naphthoquinone (plumbagin). We also tested gene expression of *nfrA* and *rrpB* in response to riboflavin and its derivatives, flavin mononucleotide (FMN) and flavin dinucleotide (FAD). Growing cultures of C. jejuni 11168h and its mutants (mid-log OD_600_, ∼0.45) were treated with the compounds, and gene expression at 15 min and 40 min was determined by RT-qPCR relative to the control after normalization using *gyrA* (Fig. 4).

In the wild-type strain the expression of *mdaB* and *rrpA* was greatly influenced by treatment with quinone compounds. The greatest effect was observed after treatment with 2-hydroxy-1,4-naphthoquinone; *mdaB* expression was increased by ∼9.6-fold, and that of *rrpA*, by ∼3.0-fold after 15 min (Fig. 4a), and *mdaB* expression remained significantly high (∼5-fold) after 40 min posttreatment (Fig. S2a). Interestingly, the expression of *mdaB* was significantly reduced (∼3.0-fold) in the Δ*rrpA* mutant strain at 15 min (Fig. 4b) and 40 min (Fig. S2b). Similarly, treatment of C. jejuni 11168H with exogenous flavins showed that *nfrA* gene expression was significantly increased up to ∼12-fold after 15 min of treatment, while *rrpB* expression was significantly reduced (∼2-fold) (Fig. 4c). *nfrA* expression remained significantly high after 40 min posttreatment with riboflavin and FMN (Fig. S2c). These results also suggest that *mdaB* and *nfrA* are under the regulatory control of RrpA and RrpB, respectively.

Given that 2-hydroxy-1,4-naphthoquinone and riboflavin had an effect on *mdaB*-*rrpA* and *nfrA*-*rrpB* expression, respectively, we tested their effects on C. jejuni growing cultures; optical density (OD_600_) was monitored for 135 min after the addition of 100 μM 2-hydroxy-1,4-naphthoquinone (Fig. 4d) and 500 μM riboflavin (Fig. 4e). Addition of 2-hydroxy-1,4-naphthoquinone led to a temporary bacteriostatic effect and reduction of the maximum optical density of strains Δ*rrpA*, Δ*rrpAB*, and Δ*mdaB* compared to the wild type (Fig. 4d); the addition of riboflavin had a modest effect on the wild-type strain, but the largest effect was observed on the Δ*nfrA* mutant strain. As OD can be affected by cell morphology, we also determined viability by CFU at 60 min and 120 min after treatment with 2-hydroxy-1,4-naphthoquinone (Fig. 4f) or riboflavin (Fig. 4g). These results were in line with the optical density measurements. We did not observe any effect on the *nfrA* mutant strain at a lower concentration (100 μM) of riboflavin.

### MdaB and NfrA reduce quinones and flavins, respectively.

**FIG 5:**
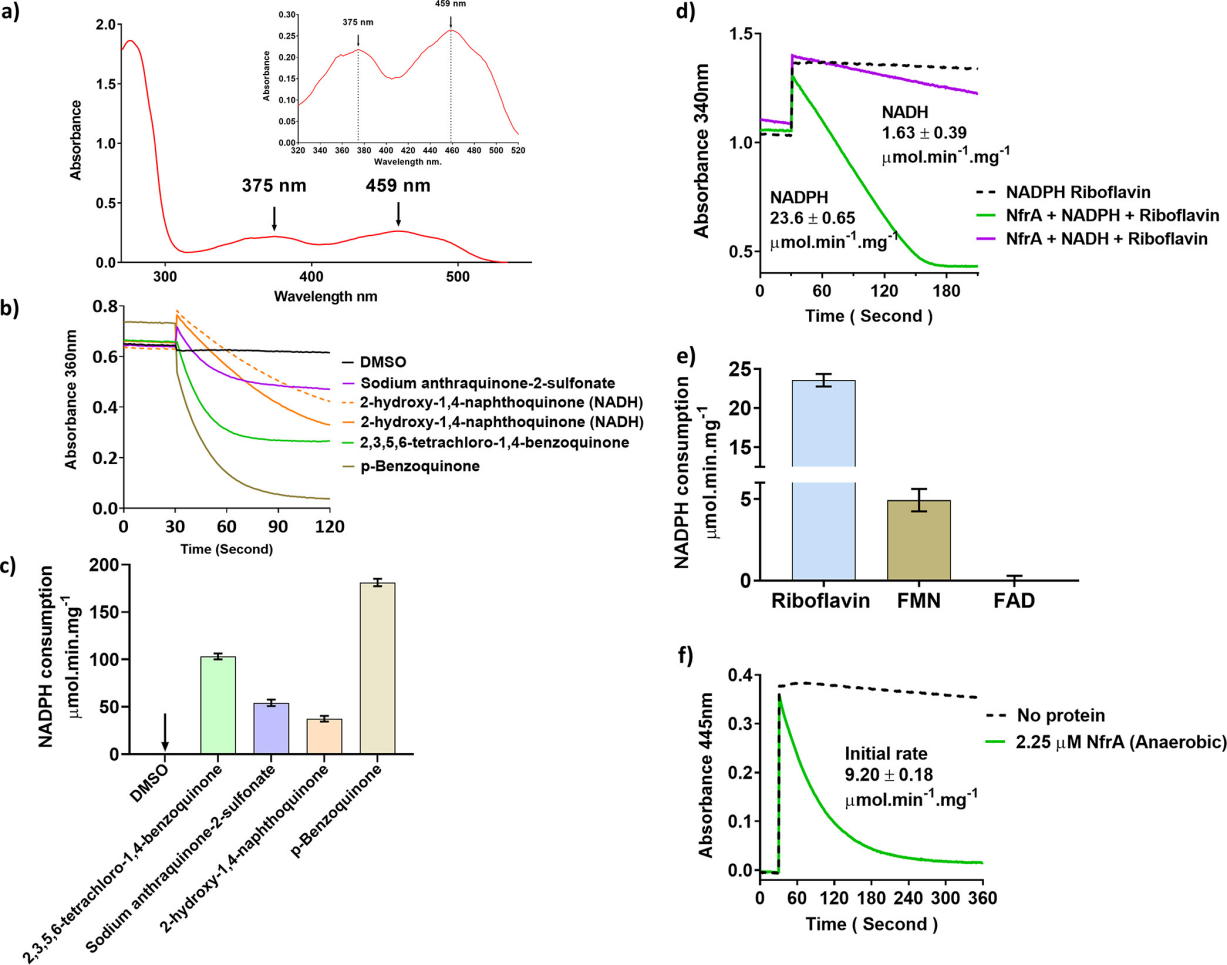
Reductase activity of purified recombinant MdaB and NfrA. (a) UV-visible spectrum of MdaB showing characteristic flavin cofactor absorbance peaks; inset shows the magnified view of the absorption spectra. (b) Traces of quinone reductase assay with purified MdaB_his6_ and NADPH (average of triplicates). (c) Specific activity of MdaB_his6_ with quinone substrates calculated from initial rate (arrow indicates <0). (d) Riboflavin reductase activity of purified NrfA_his6_ showing preference for NADPH over NADH. (e) Specific activity of NrfA_his6_ with flavin substrates and NADPH. (f) Direct measurement of riboflavin reduction by NrfA_his6_ and NADPH anaerobically by following the riboflavin absorbance maximum of 445 nm. All assays were performed at pH ∼7.5.

We conducted a conserved-domain search on the C. jejuni MdaB and NfrA proteins; MdaB contains a flavodoxin-like fold (Cl00438), while NfrA is a member of a large family that shares a Rossmann-fold NAD(P)H/NAD(P)(+) binding (NADB) domain (Cl21454). MdaB and NfrA were predicted to be a putative NAD(P)H-quinone reductase and NAD(P)H-flavin reductase, respectively; we therefore tested the reductase activity of purified recombinant C. jejuni MdaB and NfrA in the presence of quinones and flavins (riboflavin, FMN, and FAD), respectively. Purified MdaB_his6_ showed a flavin absorption spectrum with peaks at 454 nm and 379 nm, characteristic of a FAD/FMN cofactor protein (Fig. 5a). The isoalloxazine ring system within flavins generates a yellow color that is also responsible for light absorption in the UV and visible spectral range such as that observed for MdaB_his6_ (25, 32, 33). The reductase activity of MdaB was determined by monitoring the oxidation of NADPH in the presence of quinone substrates. Specific activities were determined in the range of 37 to 181 μmol min^−1^ mg^−1^ protein (Fig. 5b and c). Note that due to interference of substrates at 340 nm, 360 nm was used in these assays, and the extinction coefficient for NADPH at 360 nm was determined as 4.61 l mmol^−1^ cm^−1^. We also found that MdaB had NADPH oxidase activity under atmospheric oxygen conditions; therefore all assays were performed anaerobically (Fig. S3a). Some of the quinones tested were incompatible with the assay, due to insolubility in aqueous buffer (Fig. S3b); scans of all the quinone compounds tested (Fig. S3c) and controls (Fig. S3d) are presented in the supplemental material.

The flavin reductase activity of NfrA was determined by measuring the oxidation of NAD(P)H in the presence of the flavin substrates riboflavin, FMN, and FAD. The specific activity with riboflavin was determined as 23.6 and 1.63 μmol min^−1^ mg-^1^ protein with NADPH and NADH as the electron donor, respectively. This was indicative of NfrA’s preference for NADPH over NADH (Fig. 5d). NfrA-dependent reduction of FMN and FAD with NADPH was also determined. NfrA had some activity toward FMN (4.9 μmol min^−1^ mg^−1^, 4.8-fold lower than riboflavin) but no significant activity with FAD (Fig. 5e and Fig. S3e). Riboflavin reduction by NrfA in the presence of NADPH was monitored directly by performing the assay anaerobically and following the absorbance maximum of riboflavin (445 nm). A specific activity of 9.20 μmol min^−1^ mg^−1^ protein was calculated from the determined extinction coefficient for riboflavin at 445 nm of 2.13 liters mmol^−1^ cm^−1^ (Fig. 5f). No activity was detected with either NAD^+^ or NADP^+^ (Fig. S3f). Spectrum scans performed pre- and postassay to confirm the quantitative reduction of riboflavin are presented in the supplemental material (Fig. S3g).

### RrpA_his6_ and RrpB_his6_ are posttranslationally modified *in vitro* by treatment with redox cycling agents.

MarR-like transcription regulators utilize a redox sensing cysteine (Cys) residue “redox-switch” for their activity. We hypothesized that the same mechanism is employed by RrpA and RrpB. Protein sequence analysis revealed that RrpA has four Cys residues (Cys8, Cys13, Cys33, and Cys113), and RrpB has one Cys residue (Cys8) within the protein.

**FIG 6:**
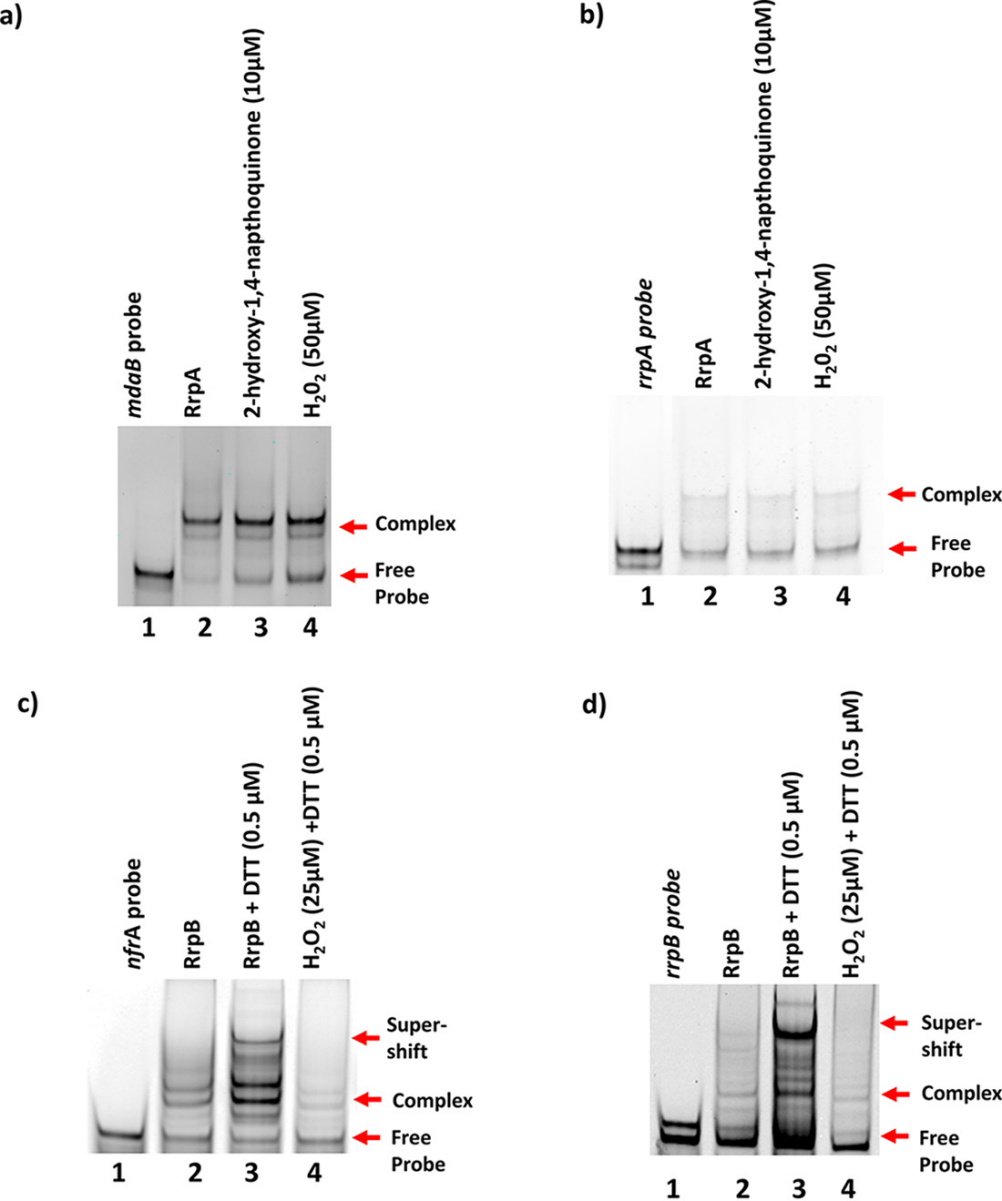
The effect of redox cycling compounds on the protein-DNA complex. EMSA was used to test the effect of 10 μM 2-hydroxy-1,4-naphthoquinone and 50 μM H_2_O_2_ on 0.05 μg of RrpA_his6_ binding to its DNA substrates *mdaB* (a) and *rrpA* (b) and the effect of H_2_O_2_ on 0.05 μg of RrpB_his6_ binding to its substrates *nfrA* (c) and *rrpB* (d). Binding is indicated by shift of band (arrows). IRDye 800 DNA fragments were used.

We tested the effects of redox cycling compounds on recombinant RrpA_his6_ and RrpB_his6_ binding to their DNA substrates by EMSA (Fig. 6). Treatment of RrpA_his6_ with 10 μM 2-hydroxy-1,4-naphthoquinone or 50 μM H_2_O_2_ did not affect its ability to form a complex with its DNA substrates, *mdaB* (Fig. 6a) and *rrpA* (Fig. 6b). Our RNA-Seq and RT-qPCR results indicated that RrpB is a negative regulator of *nfrA* and *rrpB*; considering that the environment within the cell is maintained in a reduced state, EMSAs were performed in the presence of a reducing agent (dithiothreitol [DTT]) to mimic this condition *in vitro*. Interestingly, RrpB_his6_ formed a higher complex (supershift) with its substrates in the presence of DTT (Fig. 6c and d), and the addition of 25 μM H_2_O_2_ attenuated this protein-DNA complex (Fig. 6c and d). This suggested that the derepression mechanism of RrpB is mediated by redox compounds.

We investigated whether RrpA and RrpB Cys residues are modified after treatment with redox compounds *in vitro*. Recombinant proteins were incubated with 50 μM 2-hydroxy-1,4-napthoquinone or H_2_O_2_ on RrpA_his6_ and H_2_O_2_ on RrpB_his6_ at room temperature for 30 min, and the proteins were analyzed by liquid chromatography mass spectrometry (LC-MS/MS). The total ion current (TIC) of the peptides was used to compare the treated samples to the untreated samples (Table 2).

**TABLE 2:** PTM identification following database searching and manual verification of the matched fragmentation spectra for RrpA_his6_ and RrpB_his6_ proteins^*a*^

Sample	Peptide	*m*/*z*	Charge	Mass (Da)	Residue(s)	Total ion current (TIC) intensity
RrpA (control)	^4^ENSPC_Dha_NFEEC_diox_GFNYTLALINGK^25^	821.03	3	2,460.1	Cys8; Cys13	208,300
	^28^MSILYC_Dha_LFR^36^	556.30	2	1,110.6	Cys33	204,200
	^28^M**_ox_**SILYC_Dha_LFR^36^	564.30	2	1,126.6	Met28; Cys33	110,600
RrpA (2-hydroxy-1,4-naphthoquinone)	^4^ENSPC_Dha_NFEEC_diox_GFNYTLALINGK^25^	821.03	3	2,460.1	Cys8; Cys13	1,119,000
	^28^MSILYC_Dha_LFR^36^	556.30	2	1,110.6	Cys33	877,900
RrpA (H_2_O_2_)	^4^ENSPC_Dha_NFEEC**_diox_**GFNYTLALINGK^25^	821.03	3	2,460.1	Cys8; Cys13	849,400
	^28^M**_ox_**SILYC_Dha_LFR^36^	564.30	2	1,126.6	Met28; Cys33	259,400
RrpB (control)	^4^YHSLC_sulfdiox_PIETTLNLIGNK^20^	990.48	2	1,979	Cys8	37,140
RrpB (H_2_O_2_)	4YHSLC**_sulfdiox_**PIETTLNLIGNK20	990.48	2	1,979	Cys8	82,780

a The complete data set is presented in Table S2.

Analysis of RrpA_his6_ treated with 2-hydroxy-1,4-napthoquinone or H_2_O_2_ showed a peptide with a mass peak of 2,460.1 Da and an *m/z* of 821.03^3+^; an assigned loss of 34 Da was detected on Cys8, corresponding to a dehydroalanine (Dha) modification, and the second modification was assigned with the addition of 32 Da, a di-oxidation (sulfinic acid [Cys-SO_2_H]) of Cys 13. A doubly charged peptide with a mass peak of 1,110.6 Da and an *m/z* of 556.30^2+^ was also detected in RrpA_his6_ treated with 2-hydroxy-1,4-napthoquinone; this peptide contained a single Cys residue with a matched loss of 34 Da, confirmed as a Dha modification on Cys33. RrpA_his6_ treated with H_2_O_2_ showed a peptide with a mass peak of 1,126.6 Da and an *m/z* of 564.30^2+^, which was confirmed as a Dha modification on Cys33 and a single oxidation on Met28. Dha is a desulphurization event with the potential to destabilize protein three-dimensional structure by disruption of disulfide bond formation (34). Sulfinic acid (Cys-SO_2_H) is stable and forms disulfide bonds with nearby Cys thiol groups, mediated by ROS and oxidants; both Dha and sulfinic acid modifications are reversible (35). Cys113 in RrpA_his6_ was not matched, as the tryptic peptide contained only three residues and was below the lower mass-to-charge fragmentation window set in the MS method.

Analysis of RrpB_his6_ treated with H_2_O_2_ identified a peptide with a mass peak of 1,978.95 Da and an *m/z* of 990.48^2+^. This was identified as an addition of 64 Da and confirmed as an irreversible (35) sulfur dioxide modification (thiosulfonic acid [Cys-SO_2_-SH]) at Cys8. Thiosulfonic acid results from overoxidation of Cys and is a unique by-product of degraded Cys-S-SO_2_-Cys. Degradation of this disulfide bond also produces a Dha modified cysteine (Fig. S4).

Modifications to peptides in the untreated samples were also detected, possibly generated upon sample preparation. Posttranslational modification (PTM) peptides with a lower TIC than that of the control were also detected in the treated samples, as well as possible undesired nonfunctional amino acids. A full identification table and peptide intensity values are presented in Table S2.

**FIG 7:**
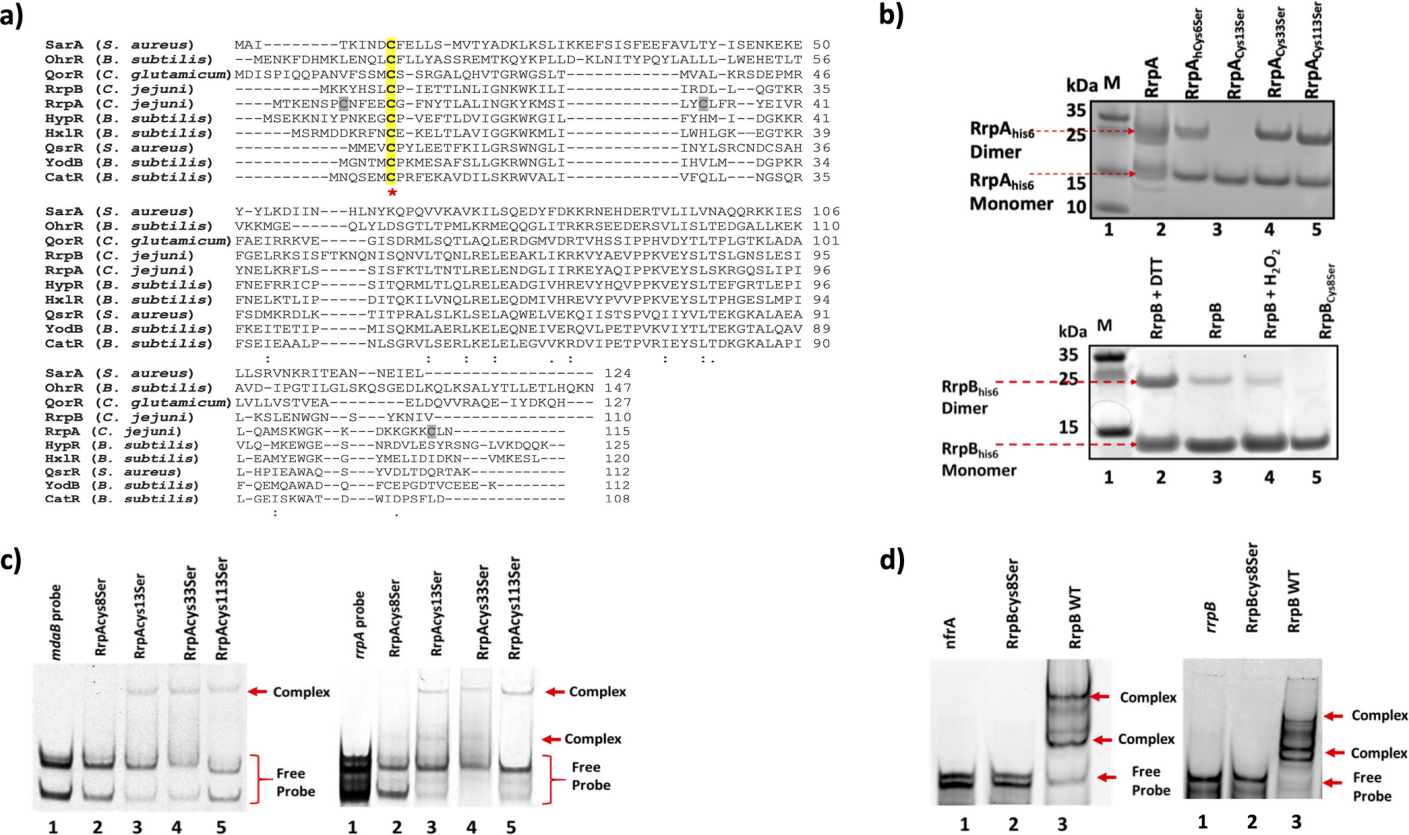
Identification of conserved cysteine residues in RrpA and RrpB proteins. (a) Sequence alignment of RrpA and RrpB with other MarR family transcriptional regulators. (b) RrpA_his6_ and RrpB_his6_ and their variants were subjected to nonreducing SDS-PAGE analysis. (c) RrpA and (d) RrpB variants with mutation to cysteine residues were generated, and EMSAs were conducted to determine their role in DNA substrate binding. Approximately 0.05 ng of RrpA and 0.05 ng of RrpB variants in the presence of 50 nM DNA substrate were used (the arrows indicate protein-DNA substrate complexes). Clustal Omega was used for multiple sequence alignment. In panel a, the yellow highlight with the asterisk indicates conserved Cys across MarR family transcription regulators. The gray highlight indicates nonconserved Cys in RrpA.

Sequence alignment (36) to other MarR family transcription regulators revealed conserved Cys residues in RrpA, Cys13, and RrpB, Cys8 (Fig. 7a). To explore the role of Cys residues in RrpA and RrpB further, we generated variants by substituting Cys with serine—RrpA_Cys8Ser_, RrpA_Cys13Ser_, RrpA_Cys33Ser_, RrpA_Cys113Ser_, and RrpB_Cys8Ser_. Nonreducing SDS-PAGE was used to analyze migration of RrpA_his6_, RrpB_his6_, and their variants. RrpA_his6_ and its variants, with the exception of RrpA_Cys13Ser_, migrated at the size of an RrpA dimer (Fig. 7b). RrpB_his6_ reduced (0.5 μM DTT) also migrated at the size of an RrpB dimer; however, RrpB_his6_ oxidized (50 μM H_2_O_2_) and RrpB_Cys8Ser_ both migrated at the size of a monomer (Fig. 7b). These results indicate that the conserved Cys residues in both proteins are critical for the dimeric forms.

The ability of RrpA_his6_ and RrpB_his6_ variants to bind DNA substrates was tested by EMSA. All RrpA variants formed protein-DNA complexes (Fig. 7c), with the exception of the nonconserved RrpA_Cys8Ser._ Similarly, RrpB mutation to the conserved sole Cys residue, Cys8, resulted in the inability of RrpB_Cys8ser_ to form protein-DNA complexes (Fig. 7d).

## DISCUSSION

We have unraveled the roles of two novel reductases, MdaB (modulator of drug activity B) and NfrA (Cj1555c), that contribute to the survival and persistence of C. jejuni. *mdaB* and *nfrA* are under the control of RrpA and RrpB, respectively, which are members of the MarR family of DNA binding proteins. RrpA is highly conserved among C. jejuni strains, while RrpB is predominant in the livestock-associated MLST clonal complex (10, 37) and is prevalent in strains isolated from humans. In other organisms, including *Helicobacter* spp., MdaB is a flavin-dependent NADPH-quinone reductase that has been suggested to fully reduce quinones and subsequently prevents the generation of the highly reactive semiquinones (38–40). NfrA is an NADPH-flavin reductase, and in other bacteria, flavin reductases have been shown to have a role in iron bioavailability and maintain a supply of flavin cofactors to proteins involved in cell homeostasis (41), but given that flavins are also redox active and given the control of *nfrA* by the redox responsive regulator RrpB, it seems more likely that NfrA reduces flavins; this potentially prevents generation of toxic semiquinones.

Organisms with a complex lifestyle such as C. jejuni often possess MarR orthologs or paralogs, which regulate genes in response to stress, including degradation of harmful phenolic compounds (2, 30, 42). MarR homologs are usually in genomic loci that are composed of divergently oriented genes encoding the transcriptional regulator and the gene(s) under its control (43). Interestingly, *mdaB* and *nfrA* are located divergently oriented upstream of *rrpA* and *rrpB*, respectively. Analysis of DNA substrate interaction assays showed that RrpA and RrpB bind sequences with inverted repeats (IRs) upstream of the translation start site of themselves and their target genes on the intergenic regions of the DNA, a common feature of this regulator family (44, 45). The identified protected regions led us to speculate that it is likely that the binding sites for RrpA are close enough to the promoter regions of *mdaB* and *rrpA* for the RNA polymerase to initiate transcription; in contrast, RrpB binding sites are distal from the RNA polymerase binding site, and the RrpB repression mechanism operates by destabilizing the DNA open complex (43).

In C. jejuni, *mdaB* was reported to have a role in oxidative stress; yet the specific role for its product has been elusive (3, 46, 47). We present evidence to show that both *mdaB* and its regulator RrpA respond to quinones. The redox-active quinone compounds can be competitively reduced to hydroquinone via a two-electron mechanism by NADPH-quinone reductases (33, 38, 48). Semiquinone radicals are cytotoxic due to their ability to react with molecular oxygen and in turn generate superoxide radicals (23). The two-electron transfer pathway of quinones produces quinols; this two-step pathway minimizes cellular damage due to the inability of quinols to cause oxidative stress. In Escherichia coli, quinols have been shown to lower the levels of superoxide ions in the cell membrane (49). Our results indicate that C. jejuni MdaB is a quinone reductase with broad substrate specificity, and treatment of C. jejuni Δ*mdaB* and Δ*rrpA* mutants with quinone showed reduction of growth compared to the parental strain. In both the external environment and in the host and the gut, many organisms synthesize toxic quinones (50) and derivatives that form core constituents of many antimicrobial compounds (51–53). It is plausible that C. jejuni would come into contact with these compounds. We propose that MdaB contributes to the protection of C. jejuni from production of semiquinones by competing with the quinone one-electron reduction pathway, as also suggested by Palyada et al. (26).

The NADPH-flavin reductase described here is the product of the *nfrA* gene; it belongs to the family of flavin reductases that were first isolated from luminous marine bacteria (54, 55) and from human erythrocytes (56). The fact that it took a high concentration of riboflavin to have an effect on growing the C. jejuni Δ*nfrA* mutant strain and that NfrA reduces riboflavin at a much higher rate than its 5′ phosphorylated form (FMN), which is produced by riboflavin kinase within the cell suggests that unidentified exogenous free flavins are its natural substrates. A recent study has identified a chemical analogue of riboflavin that has antimicrobial activity. Roseoflavin (8-demethyl-8-dimethylamino-riboflavin) is a broad-spectrum antibiotic naturally produced by *Streptomyces* spp. and has been shown to be effective against several bacterial species and protozoans (57). Import of this compound was shown to be mediated by riboflavin transporters, and cellular targets for roseoflavin included FMN riboswitches and flavoproteins (58, 59). Many bacteria employ FMN riboswitches, and all cells depend on the activity of flavoproteins for homeostasis, so inhibition of these systems can be detrimental to the cell. Like other flavins, roseoflavin has the potential to generate highly toxic reactive semiquinones (60). Thus, we speculate that NfrA reduces flavin compounds via the two-electron reductant pathway, protecting C. jejuni against reactive flavo-semiquinones and flavin analogues.

The distribution of *mdaB* and *nfrA* in the complete genome sequences of 374 Campylobacter spp. and 253 *Helicobacter* spp. was studied (Table. S3a and b). *mdaB* was present in all 627 genome sequences analyzed despite the absence of *rrpA*. *nfrA* was absent in the *Helicobacter* sp. sequences analyzed, while in the majority Campylobacter spp., *nfrA* was predicted as incomplete. In the majority of Campylobacter sp. sequences that lacked a complete *nfrA*, *rrpB* was either absent or partially present. This suggested that *mdaB* is important in both organisms. Furthermore, C. jejuni strains that are less frequently isolated in human infections lack a complete *nfrA*; this indicates that these strains have an as yet unidentified mechanism of flavin reduction.

**FIG 8:**
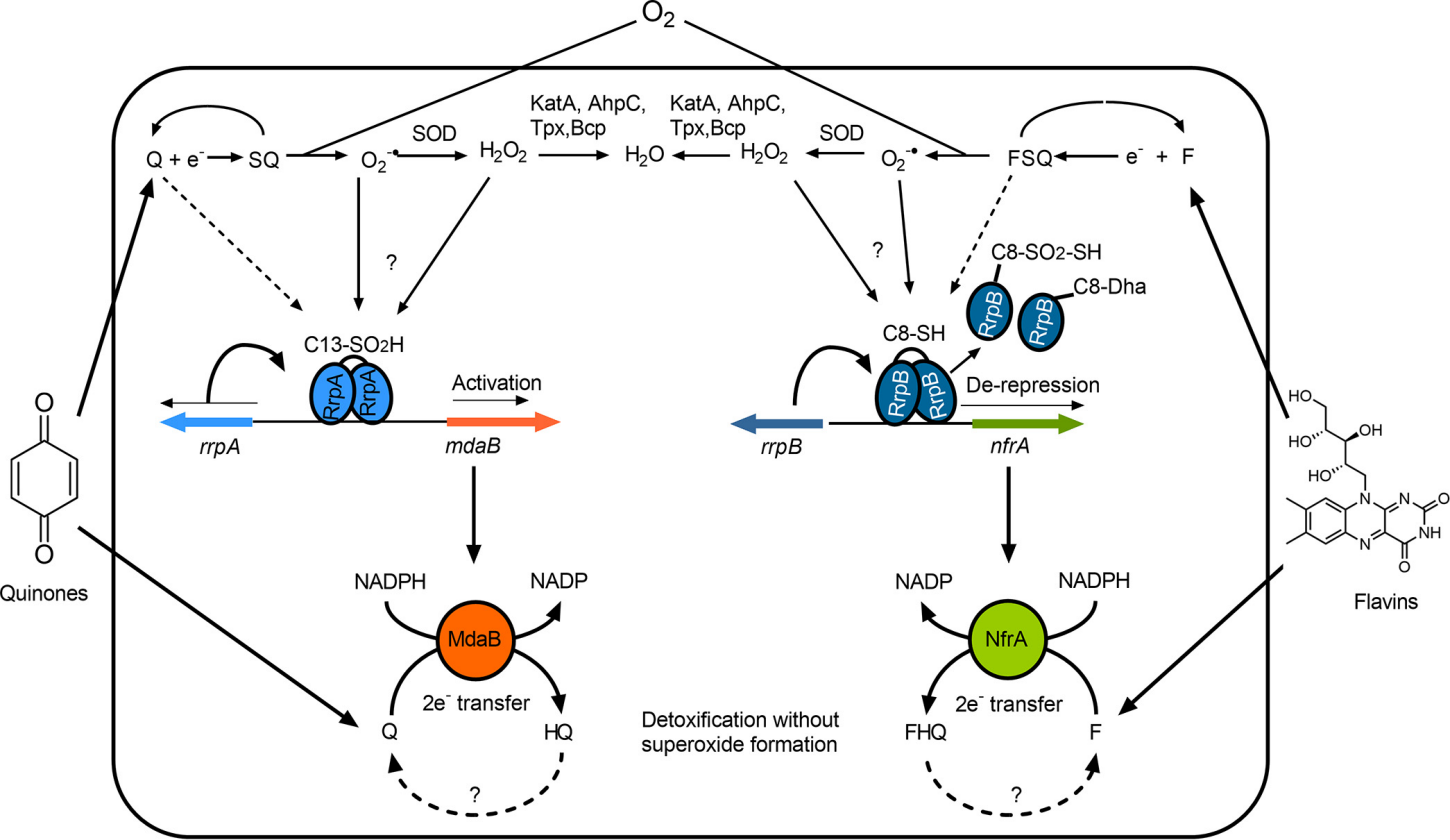
Model of the activation of expression and function of MdaB and NfrA by RrpA and RrpB. Structurally diverse quinones (Q) and flavins (F) produced by other bacteria in the intestinal microbiota or other environments encountered by C. jejuni enter the cell, where they easily abstract single electrons from the flavins or metal centers of cellular redox enzymes (62) to form partially reduced semiquinones (SQ) or flavo-semiquinones (FSQ). In the presence of molecular oxygen, these can lead to the formation of toxic superoxide and other ROS (hydrogen peroxide), which may be dealt with by superoxide dismutase, catalase, and thiol peroxidases (AhpC, Tpx, and Bcp). Elevated ROS leads to oxidation of Cys13 of RrpA and Cys8 of RrpB, although the exact species which effects this oxidation is not known. It is also possible that Q/F-Cys adduct formation (by Michael addition) occurs (dashed lines). Oxidized/modified RrpA C13 to C13-SO_2_H leads to an increased activation of the divergently transcribed gene *mdaB*, leading to production of MdaB, which is a quinone reductase that can fully reduce quinones to quinols (HQ) by two-electron transfer from NADPH. RrpB is a negative regulator of the divergently transcribed *nfrA* gene, encoding an NADPH-dependent flavin reductase (NfrA). We presume that oxidation/modification of RrpB C8-SH produces a C8-SO_2_-SH and C8-Dha (dehydroalanine) which destabilizes the dimer and, in turn, leads to the derepression of *nfrA* expression, allowing NfrA to catalyze the formation of fully reduced flavin hydroquinone (FHQ). The two-electron transfer catalyzed by both MdaB and NfrA avoids the semiquinone-mediated production of ROS. It is not known if or how cycling back to the oxidized forms occurs. This mechanism leads to a decrease in the toxic threat from exogenous quinones and flavins, but it comes at the expense of a drain on the cellular NADPH pool.

RrpA and RrpB share conserved cysteines (Cys) with other MarR family transcription regulators, and *in vitro* posttranslational modification analysis indicated that these Cys residues are modified by oxidizing compounds. Our results showed the importance of the conserved Cys13 in RrpA for dimerization but not for DNA binding. Studies have shown that MarR transcription regulators form homodimers, and their transcriptional regulation ability, but not their DNA binding ability, can depend on the dimeric form (30, 61). We speculate that RrpA Cys13 is most likely important for transcription regulation. Mutation to the nonconserved RrpA Cys8 led to its inability to bind DNA substrate, possibly due to structure destabilization. Further work will be needed to investigate the role of Cys 8 in RrpA structure. Similarly, RrpB_Cys8Ser_ was unable to dimerize or form protein-DNA complex with its substrates, and interestingly, treatment with a redox cycling compound had the same effects as the mutation to RrpB Cys8. *In vitro* PTM indicated that RrpB Cys8 is modified to the irreversible thiosulfonic acid; we propose that the derepression mechanism of RrpB is due to oxidation modification to Cys8. In E. coli, the SoxRS regulatory system detects and is oxidized by redox cycling compounds such as quinones, and one of the members of the SoxRS regulon is *mdaB* (62). From our work, it is now apparent that although C. jejuni has long been known to lack *soxRS* genes, the RrpA and RrpB system described here is at least partly functional analogously. A model for how we believe the regulatory proteins and protective enzymes allow C. jejuni to combat quinone- and flavin-mediated oxidative stress is shown in Fig. 8.

We also noted that other genes were differentially regulated in our regulator mutants, and although the expression levels were not necessarily related to functional importance, it is possible that mutations in *rrpA* and *rrpB* affect other pathways that are not investigated here, in particular, ribosomal genes, autotransporters, and amino acid metabolism. However, it is interesting that expression of *leuA* was increased in the *rrpAB* double mutant. The dehydratase enzymes (LeuCD) of the branched-chain amino-acid biosynthesis pathway contain labile Fe-S clusters and have long been known to be targets for ROS damage in E. coli (63), and perhaps *leuA* upregulation reflects this.

### Conclusion.

MdaB and NfrA are reductases specific to their substrates and are under the control of the redox regulators RrpA and RrpB, respectively. MdaB catalyzes the reduction of quinones, while NfrA is a flavin reductase; thus, it is possible that both enzymes contribute to C. jejuni protection against potential reactive semiquinone species. We propose that possession of these enzymes by C. jejuni is important during its *in vivo* and *ex vivo* life cycle and could be a significant factor for the persistence and prevalence of C. jejuni in the food chain.

## MATERIALS AND METHODS

### Bacterial cultures and strains.

Bacteria were stored using Protect bacterial preservers (Technical Service Consultants, Heywood, UK) at −80°C. C. jejuni strains were streaked on blood agar (BA) plates containing Columbia agar base (Oxoid) supplemented with 7% (vol/vol) horse blood (TCS Microbiology, UK) and Campylobacter selective supplement (Oxoid) and were grown at 37°C in a microaerobic chamber (Don Whitley Scientific, UK) containing 85% N_2_, 10% CO_2_, and 5% O_2_ for 48 h. C. jejuni strains were grown on Columbia blood agar (CBA) plates for a further 16 h prior to use. Strain 11168H of multilocus sequence type (MLST) clonal complex ST-21, a hypermotile derivative of the original strain NCTC11168 that shows a higher level of cecum colonization in a chick model (8) was used in this study. Construction of mutants in which *rrpA* and *rrpB* have been inactivated to give isogenic mutants Δ*rrpA* and Δ*rrpB* has been previously described (6, 7).

### Protein expression and purification.

Recombinant RrpA, RrpB, and their variants RrpA_Cys8Ser_, RrpA_Cys13Ser_, RrpA_Cys33Ser_, RrpA_Cys113Ser_, and RrpB_Cys8Ser_ and MdaB and NfrA were cloned into pET21a^+^ plasmid using a NEBuilder HiFi DNA assembly cloning kit (New England Biolabs). The proteins were overexpressed in E. coli strain BL21(DE3). The cells were grown in LB broth containing 0.6% glycerol, 0.05% glucose, and 0.2% lactose and 100 μg/ml ampicillin at 37°C overnight. Cells were harvested by centrifugation at 4,000 × *g* for 15 min at 4°C and resuspended in buffer A (20 mM Tris-HCl, pH 8.0, 500 mM NaCl, 5% [vol/vol] glycerol, and 5 mM 2-mercaptoethanol containing EDTA-free cOmplete protease inhibitor mixture [Roche]) for lysis. Cell debris was removed by centrifugation at 10,000 × *g* for 30 min at 4°C. The supernatant was incubated with Ni-NTA agarose nickel-charged resins (Qiagen) that had been equilibrated in buffer A. The protein-bound resin was washed with buffer A containing 15 mM imidazole and eluted with buffer A containing 400 mM imidazole. The primers used to generate recombinant proteins are presented in Table S4a).

### Gene expression by RNA-Seq and bioinformatics analysis.

RNA-Seq was used to identify differentially expressed genes between wild-type strains and Δ*rrpA* and Δ*rrpB* mutants at the mid-log phase (∼6 h) of growth. C. jejuni 11168H wild type, 11168HΔ*rrpA*, 11168HΔ*rrpB*, and 11168HΔ*rrpAB* were plated on BA plates and incubated at 37°C under microaerobic conditions for 6 h; 25 ml of preincubated brucella was inoculated with C. jejuni strains at an OD_600_ of 0.1 and grown at 37°C under microaerobic conditions as described above. Transcription was stopped by RNAprotect (Qiagen). RNA was extracted using a PureLink RNA minikit (Invitrogen) following the manufacturer’s protocol. rRNA was depleted using Ribominus (Invitrogen), and libraries were prepared using TruSeq stranded mRNA (Illumina). Raw reads were obtained from an Illumina MiSeq paired-end sequencing platform. The paired-end reads were trimmed and filtered using Sickle v1.200 (64). Bowtie 2 (65) was used to map the reads against the reference sequence, C. jejuni strain 11168H (GenBank assembly number GCA_900117385.1). The Cufflinks suite (66) was used to convert annotations from GFF to GTF format, and BEDTools (67) was used to generate transcript counts per samples. Statistical analysis was performed in R using the combined data generated from the bioinformatics and meta data associated with the study (multifactorial design). An adjusted *P* value significance cutoff 0.05 and log fold change cutoff of >1.5 was used for multiple comparison.

### Real-time RT-qPCR.

Expression of genes of interest were quantified by real-time RT-qPCR and normalized against *gyrA*. A 1-μg volume of total RNA of each sample was reverse-transcribed to cDNA using the RT^2^ first strand kit (Qiagen) according to manufacturer’s protocol. Quantification of gene expression was achieved by real-time RT-qPCR using TaqMan primers and probes created by the Assay-by-Design Service of Applied Biosystems (Table. S4b). Real-time RT-PCR was performed in 96-well plates using an ABI PRISM 7300 real-time PCR system (Applied Biosystems), and the relative gene expression for the different genes was calculated from the crossing threshold (*C_T_*) value according to the manufacturer’s protocol (2^−ΔΔ^*^CT^*) after normalization using the *gyrA* endogenous control (68).

### DNase I footprinting and electrophoretic mobility shift assay.

DNase I footprinting was performed as previously described (29). Briefly, a 393-bp DNA fragment was PCR amplified with primers modified with fluorescein amidite (FAM) and hexachloro-fluorescein (HEX). Primers were used to amplify regions upstream of the translation initiation sites of *rrpA* and *mdaB* (cj1546Ffam and cj1546Rhex) and for *rrpB* and *nfrA* (cj1556Ffam and cj15556Rhex). PCR was performed for 30 cycles at the following conditions: 95°C for 60 s, 58°C for 60 s, and 72°C for 60 s. The FAM/HEX‐labeled probes were cleaned using a QiAquick PCR purification kit (Qiagen) and quantified with a NanoDrop spectrometer (Thermo Scientific). For the DNase I footprinting assay, 500-ng probes were incubated with various concentrations (from 20 μg to 0 μg) of RrpA_his6_ or RrpB_his6_ in a total volume of 40 μl in buffer containing 30 mM potassium glutamate, 1 mM dithiothreitol (DTT), 5 mM magnesium acetate, 2 mM CaCl_2_, 0.125 mg/ml bovine serum albumin (BSA), and 30% glycerol in 10 mM Tris HCl, pH 8.5). After incubation for 20 min at 25°C, 10 μl of 0.02 unit of DNase I (NEB) was added to the binding reaction and incubated for a further 5 min at 25°C. The DNase I was inactivated by incubating the reaction at 74°C for 10 min. Samples were cleaned and eluted in 20 μl of distilled water (dH_2_O). The samples were run with the 3730 DNA analyzer and viewed with GeneMapper v6 (Applied Biosystems).

For EMSA, purified RrpA_his6_ (0.05 μg) was incubated with IRDye 800 DNA fragments spanning the identified binding regions upstream of the translation start sites of *mdaB* (mdaBprobe), *rrpA* (rrpAprobe), and RrpB_his6_ (0.05 μg) with *nfrA*(nfrAprobe) and *rrpB* (rrpBprobe); a 50-nm probe was used in a 20-μl reaction. An Odyssey infrared EMSA kit (LI-COR Biosciences) was used according to the manufacturer’s instructions. Samples were loaded in precast 6% Novex DNA retardation gel (Life Technologies) and run at 4°C, and gels were analyzed on a LI-COR Odyssey imaging scanner (LI-COR Biosciences). The list of primers for DNase I footprinting and probes for EMSA can be found in Table S4c.

### *In vitro* posttranslation modification analysis using LC-MS/MS.

Recombinant (1 μg) RrpA_his6_ was treated with 50 μM 2-hydroxy-1,4-napthoquinone or 50 μM H_2_O_2_, and the RrpB_his6_ was treated with 100 μM H_2_O_2_ for 30 min at room temperature. Samples were resuspended in 500 μl of 50 mM tetraethylammonium bicarbonate (TEAB; Sigma), vortexed, and centrifuged at 14,000 rpm for 1 min. Overnight trypsin digestion at 37°C was performed without the reduction and alkylation of cysteine residues to protect posttranslational modification. Samples were dried and resuspended in 40 μl of 2% acetonitrile in 0.05% formic acid, 10 μl of which was injected to be analyzed by LC-MS/MS. Chromatographic separation was performed using a U3000 UHPLC NanoLC system (Thermo Fisher Scientific). Peptides were resolved by reversed-phase chromatography on a 75-μm C_18_ Pepmap column (50 cm length) using a four-step linear gradient of 80% acetonitrile in 0.1% formic acid. Raw mass spectrometry data were processed into peak list files using Proteome Discoverer v2.2 (Thermo Fischer Scientific). The raw data file was processed and searched using the Sequest search algorithm (69) against a bespoke database containing the RrpA and RrpB protein sequences obtained from UniProt (Q0P879 [RrpA] and Q0P870 [RrpB]).

### Reductase assays.

An assay mixture consisting of 50 mM Tris-HCl, 100 mM NaCl, 10% vol/vol DMSO, 200 μM NADPH, and 2 μM MdaB was sparged with oxygen-free nitrogen for 7 min, followed by incubation at 37°C for 2 min. Absorbance was recorded at 360 nm for 30 secs before the addition of 0.2 mM substrate. NADPH consumption was recorded for a further 1.5 min. FMN and FAD were prepared in dH_2_O, while riboflavin was prepared in 10 mM NaOH; NfrA activity was determined at an absorbance of 340 nm, and NAD(P)H consumption was recorded for a further 3 min. No protein controls were performed with all substrates.

### Growth measurement.

Brucella broth was preincubated at 37°C under microaerobic conditions for 24 h. Following overnight growth on BA, the bacterial cells were subcultured in 10 ml of the preincubated broth in a 30-ml flask at an OD_600_ of 0.01 at 37°C under microaerobic conditions and grown to an OD_600_ of 0.4. Compounds were added to the respective strains at the indicated concentrations (Fig. 4), and OD_600_ readings were performed at selected time points.

### Statistics.

Statistical analyses for RNA-Seq were performed in R using the combined data generated from the bioinformatics as well as metadata associated with the study (multifactorial design). Differentially expressed genes were considered significant when the *P* value of five independent biological experiments was below 0.05. For other experiments, two-way analysis of variance (ANOVA) with a Šídák multiple-comparison test and Student’s *t* tests were performed to obtain *P* values using the software Prism (version 9, GraphPad Software, Inc.).

### Data availability.

The data that support the RNA-seq findings of this study are openly available in the Gene Expression Omnibus (GEO) under data set identifier GSE174333. Data that support the posttranslational modification finding of this study are openly available in ProteomeXchange via the PRIDE database under data set identifier PXD025924.
