# Supplementary material for: MdaB and NfrA, Two Novel Reductases Important in the Survival and Persistence of the Major Enteropathogen Campylobacter jejuni
Source: J Bacteriol. 2022 Jan 18;204(1):e00421-21. doi: 10.1128/JB.00421-21 (PMC8765430; doi:10.1128/JB.00421-21)
Supplement: Supplemental file 4 — Fig. S1 to S4 and Table S4. Download JB.00421-21-s0004.pdf, PDF file, 0.8 MB [file jb.00421-21-s0004.pdf]

MdaB and NfrA, two novel reductases important in the survival and persistence of the major enteropathogen *Campylobacter jejuni*

Running title: MdaB and NfrA Aid Survival of *C. jejuni*

Fauzy Nasher[1]*, Aidan J. Taylor[2], Abdi Elmi[1], Burhan Lehri[1], Umer Z. Ijaz[3], Dave Baker[4], Richard Goram[5], Steven Lynham[6], Dipali Singh[4], Richard Stabler[1], David J. Kelly[2], Ozan Gundogdu[1], Brendan W. Wren [1]*.

[1]Faculty of Infectious and Tropical Diseases, London School of Hygiene and Tropical Medicine, London, United Kingdom.

[2]Department of Molecular Biology and Biotechnology, University of Sheffield, Sheffield, United Kingdom.

[3]School of Engineering, University of Glasgow, Glasgow, United Kingdom

[4] Microbes in Food Chain, Quadram Institute Biosciences, Norwich Research Park, Norwich, United Kingdom.

[5]The John Innes Centre, Norwich Research Park, Norwich, United Kingdom

[6]Proteomics Facility, Centre of Excellence for Mass Spectrometry, King's College London, London, United Kingdom.

*Address correspondence to **Brendan W. Wren**, brendan.wren@lshtm.ac.uk
and **Fauzy Nasher**, fauzy.nasher1@lshtm.ac.uk.

Figure. S1:

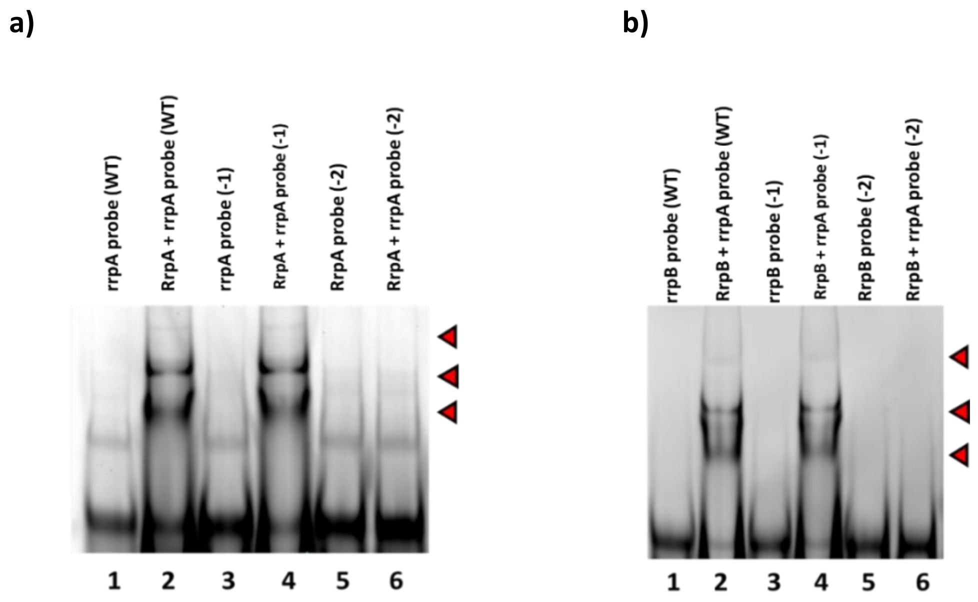

DNA fragments used:

***rrpA* WT:**
5'-
GCATATAA**TATCAT**TTTCAATACAGAAAATAAAGTAATA**TACTAT**ACTAAAGGAATGTT
AAATGA - 3'
***rrpA* (-1):**
5'-
GCATAA**TATCAT**TTTCAATACAGAAAATAAAGTAATA**TCGAGC**ACTAAAGGAATGTTAA
ATGA - 3'
***rrpA* (-2):**
5'-
GCATAA**TCGAGC**TTTCAATACAGAAAATAAAGTAATA**TCGAGC**ACTAAAGGAATGTTA
AATGA - 3'

***rrpB* WT:**
5'- TTAAAAAATATG**TTATAA**TTTTAAAAAATAAGGAT**TTATAA**TGAAAAAATAT - 3'
***rrpB* (-1):**
5'- TTAAAAAATATG**TTATAA**TTTTAAAAAATAAGGAT**CGGATC**TGAAAAAATAT - 3'
***rrpB* (-2):**
5'- TTAAAAAATATG**CGGATC**TTTTAAAAAATAAGGAT**CGGATC**TGAAAAAATAT - 3'

Figure S1: $RrpA_{his6}$ and RpB_{his6} binding specificity. EMSA was conducted to confirm specificity of $RrpA_{his6}$ and $RrpB_{his6}$ proteins to their binding sequences repectively. **a)** EMSA showing $RrpA_{his6}$ protein:DNA complex to WT DNA **(Lane 2)** that contains the inverted repeats found within RrpA binding sequence; **(Lane 4)** protein:DNA complex of $RrpA_{his6}$ and a DNA with a mutation to one of the IR motif (-1); **(Lane 6)** no protein:DNA complex formed when $RrpA_{his6}$ protein was incubated with a double mutated DNA (-2) of the IR motif sequnce. The same was repeated for $RrpB_{his6}$, **b)** $RrpB_{his6}$ forms protein:DNA complex with

WT DNA **(Lane 2)** and a single mutation to one of the palindrome motif (-1) on the DNA **(Lane 4);** but was unable to form protein:DNA complex with DNA that lacks both palindrome motif **(Lane 6)**. IRDye® 800 DNA fragments were used; the arrows indicate protein:DNA complex. DNA fragments used are shown above; bold underlined are the binding box for RrpA and RrpB; red font indicate mutations made to the binding box.

Figure. S2:
a)

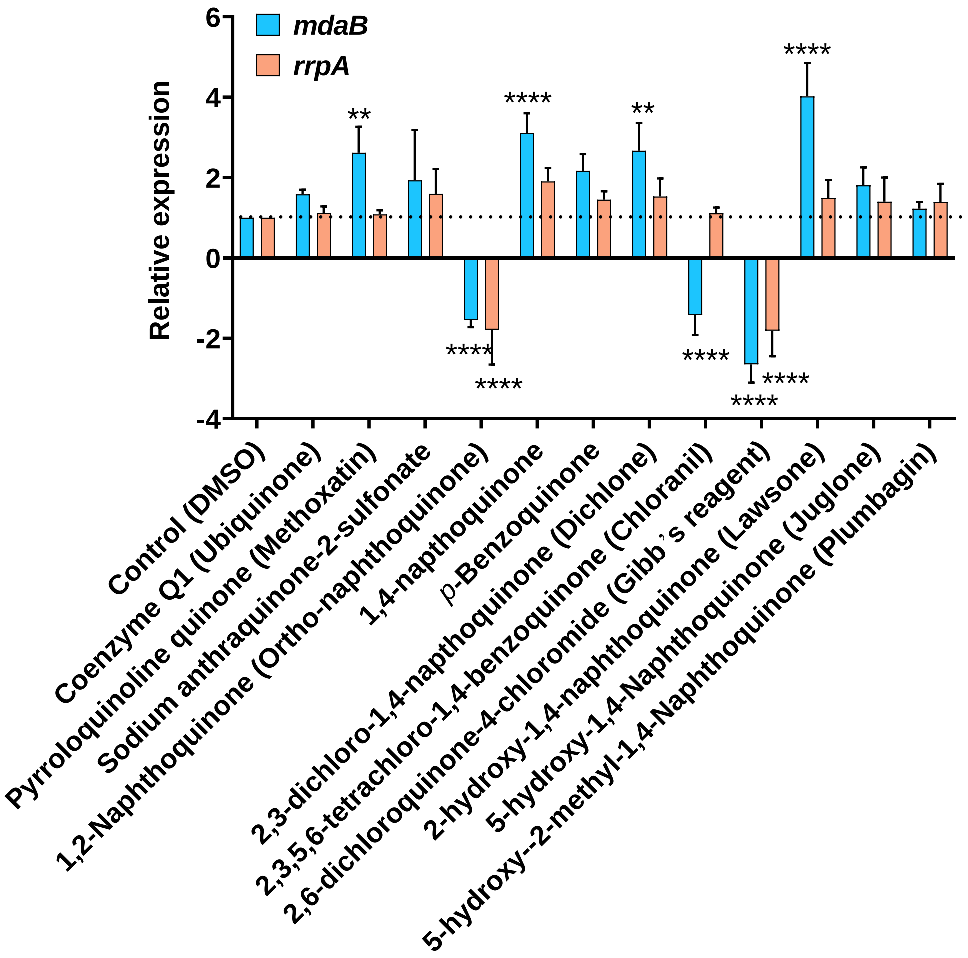

mdaB
rrpA
Relative expression
6
4
2
0
-2
-4
**
****
**
****
****
****
****
****
****
Control (DMSO)
Coenzyme Q1 (Ubiquinone)
Pyrroloquinoline quinone (Methoxatin)
Sodium anthraquinone-2-sulfonate
1,2-Naphthoquinone (Ortho-naphthoquinone)
1,4-napthoquinone
p-Benzoquinone
2,3-dichloro-1,4-napthoquinone (Dichlone)
2,3,5,6-tetrachloro-1,4-benzoquinone (Chloranil)
2,6-dichloroquinone-4-chloromide (Gibb´s reagent)
2-hydroxy-1,4-naphthoquinone (Lawsone)
5-hydroxy-1,4-Naphthoquinone (Juglone)
5-hydroxy--2-methyl-1,4-Naphthoquinone (Plumbagin)

b)

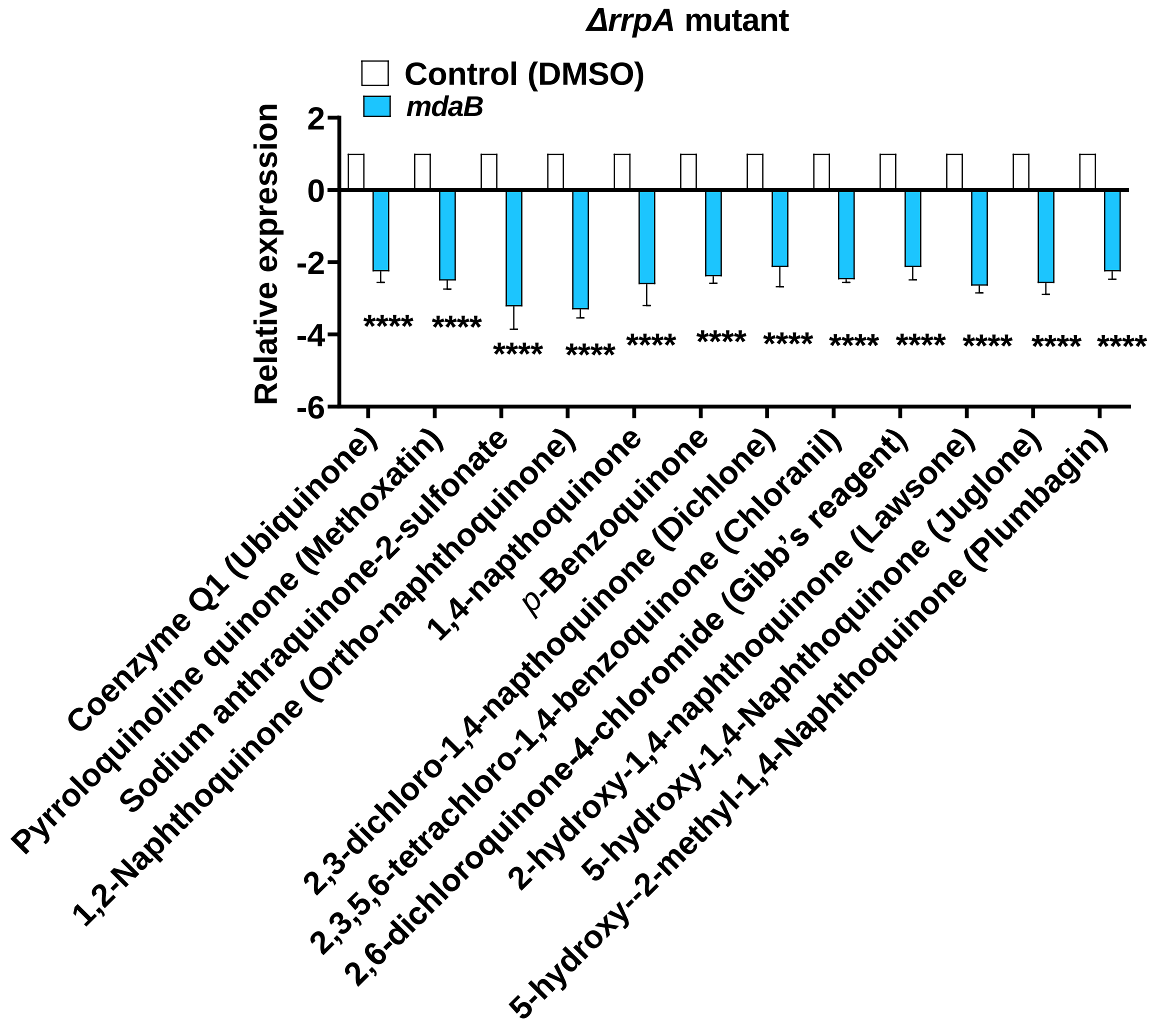
ΔrrpA mutant
Control (DMSO)
mdaB
Relative expression
2
0
-2
-4
-6
****
Coenzyme Q1 (Ubiquinone)
Pyrroloquinoline quinone (Methoxatin)
Sodium anthraquinone-2-sulfonate
1,2-Naphthoquinone (Ortho-naphthoquinone)
1,4-napthoquinone
p-Benzoquinone
2,3-dichloro-1,4-napthoquinone (Dichlone)
2,3,5,6-tetrachloro-1,4-benzoquinone (Chloranil)
2,6-dichloroquinone-4-chloromide (Gibb's reagent)
2-hydroxy-1,4-naphthoquinone (Lawsone)
5-hydroxy-1,4-Naphthoquinone (Juglone)
5-hydroxy--2-methyl-1,4-Naphthoquinone (Plumbagin)

c)

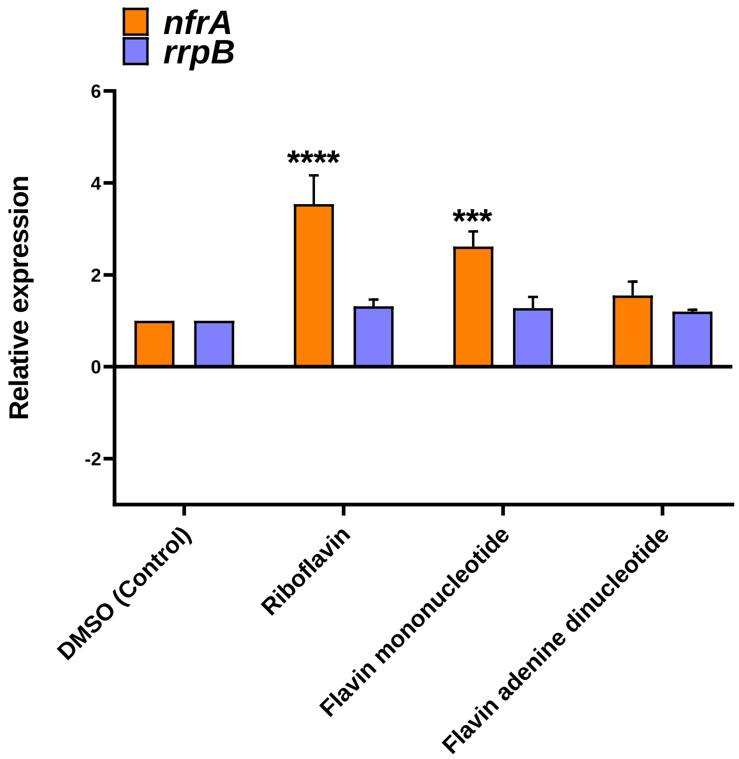

Figure. S2. Relative gene expression after treatment with quinones. Expression of (**a**) *mdaB* and *rrpA* at 40 mins after treatment with quinones; (**b**) expression of *mdaB* in the Δ*rrpA* mutant strain after 40 mins treatment with quinones; and **c)** *nfrA* and *rrpB* gene expression at 40 mins after treatment with riboflavin and derivatives. Gene expression was determined by real-time RT-PCR after treatment with 100 µM of compounds and is displayed relative to the value of the control expression, after normalization using *gyrA* expression. The values are the means of at least three separate experiments. Error bars indicate standard deviation., *$p \leq 0.05$, **$p \leq 0.01$, ***$p \leq 0.001$, ****$p \leq 0.0001$.

Figure. S3:

a)

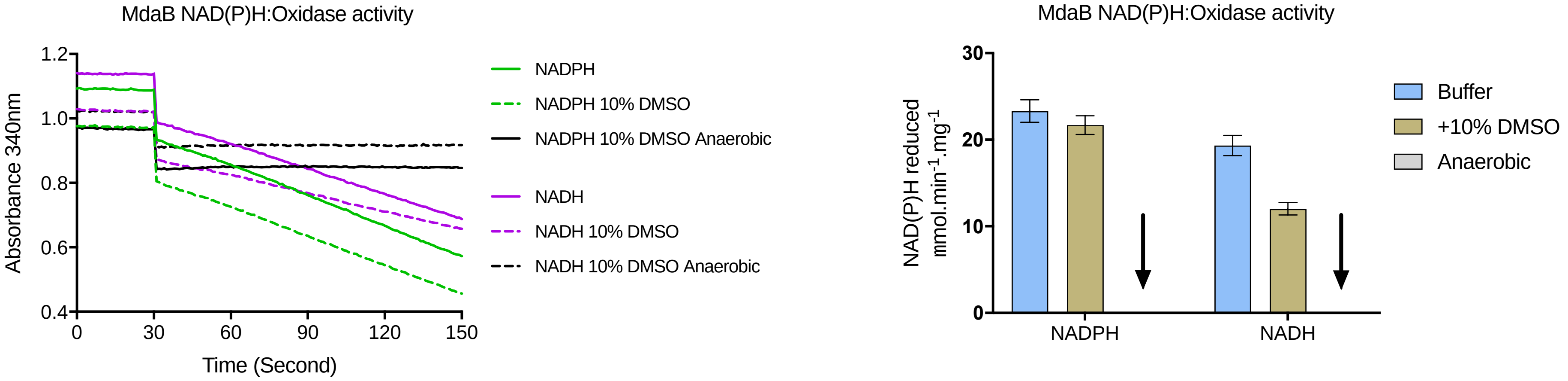

b)

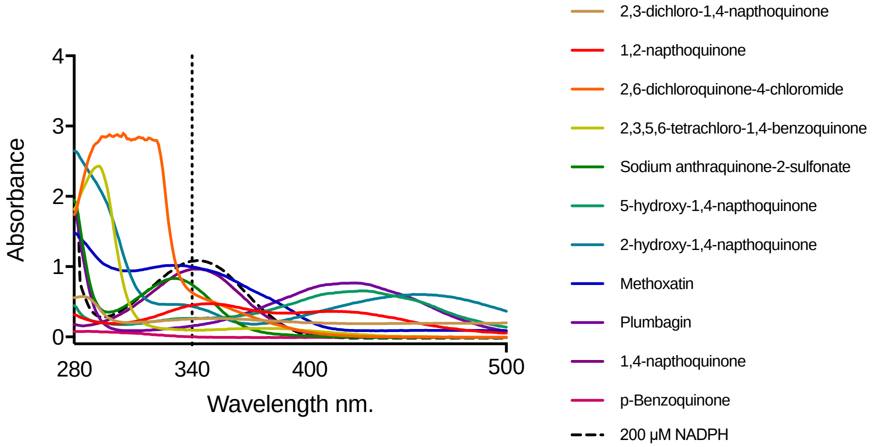

c)

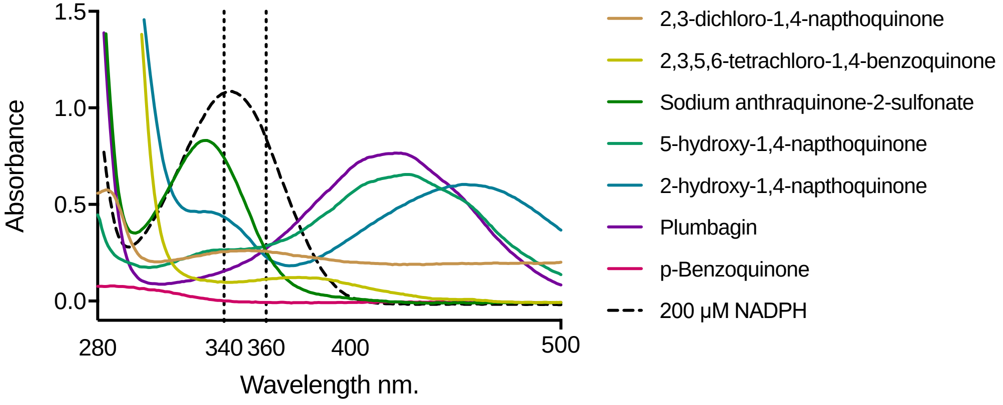

d)

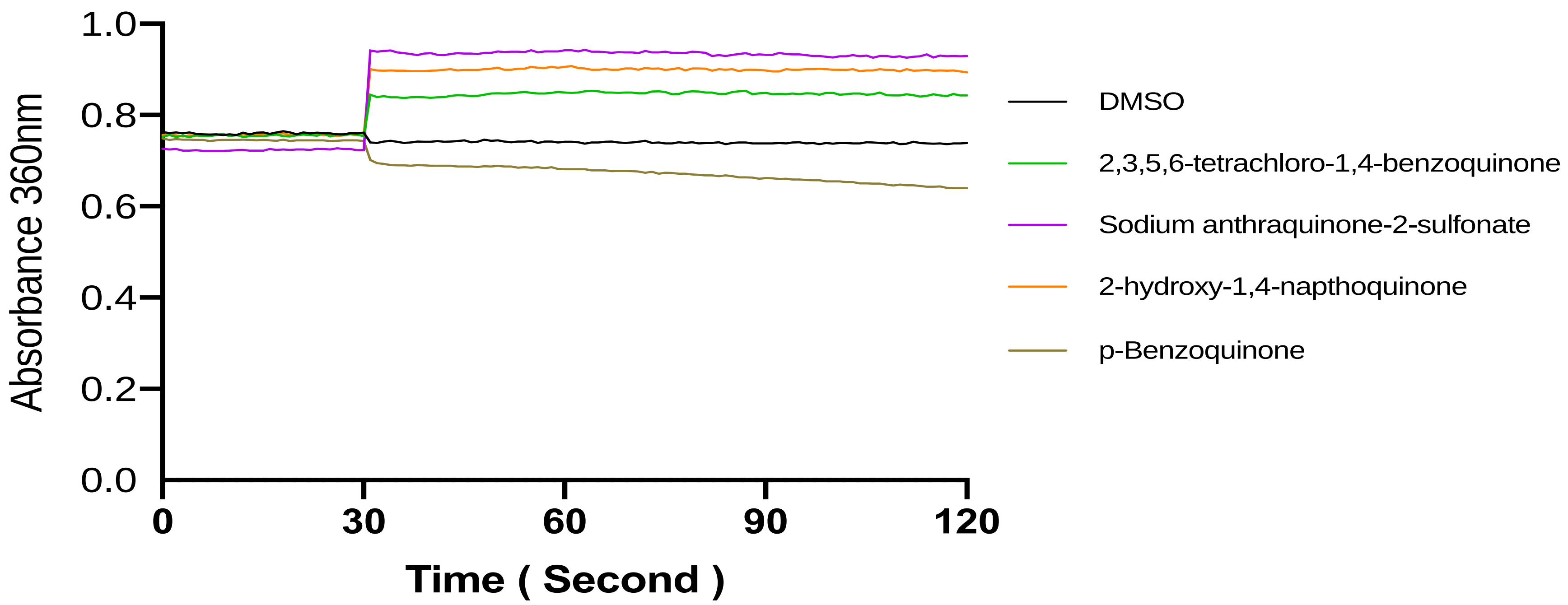

1.0
0.8
0.6
0.4
0.2
0.0
Absorbance 360nm
0
30
60
90
120
Time (Second)
DMSO
2,3,5,6-tetrachloro-1,4-benzoquinone
Sodium anthraquinone-2-sulfonate
2-hydroxy-1,4-napthoquinone
p-Benzoquinone

e)

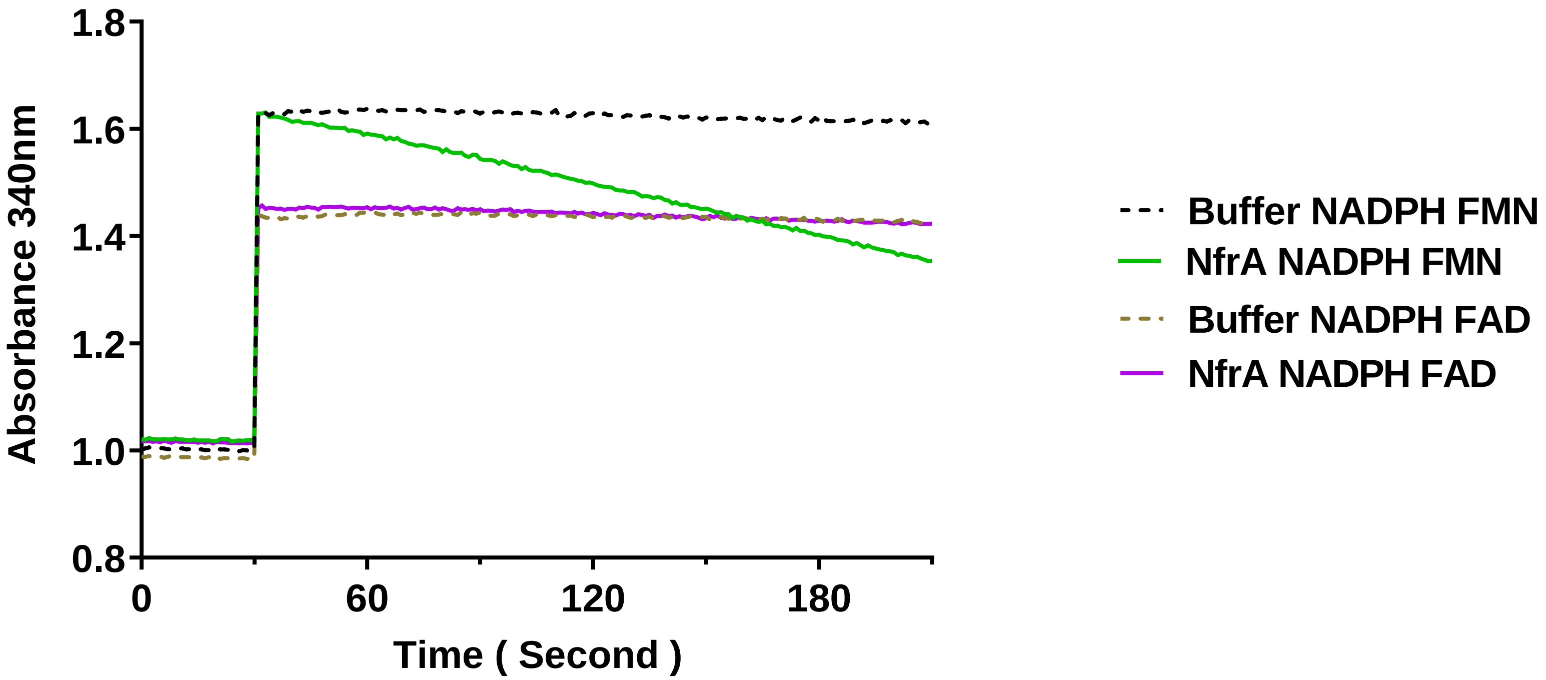

Absorbance 340nm
1.8
1.6
1.4
1.2
1.0
0.8
0
60
120
180
Time (Second)
Buffer NADPH FMN
NfrA NADPH FMN
Buffer NADPH FAD
NfrA NADPH FAD

f)

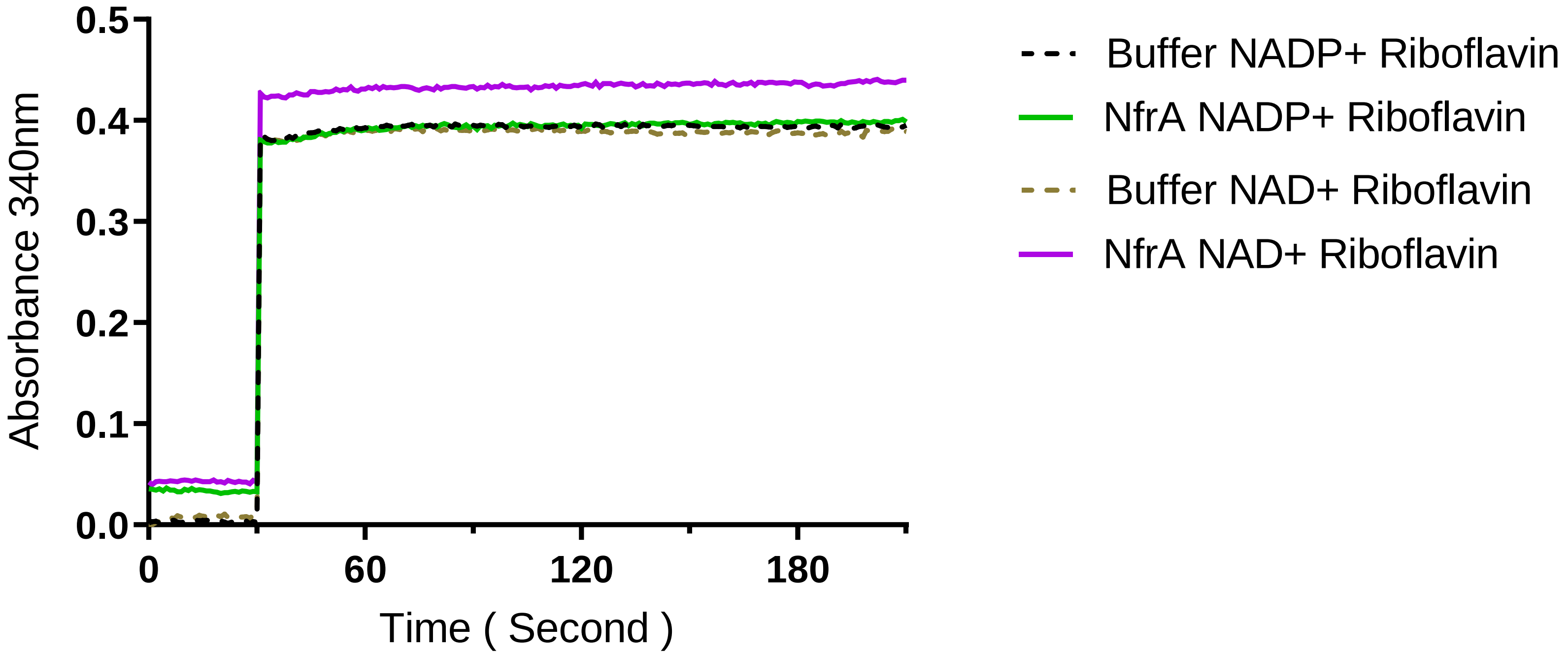

0.5
0.4
0.3
0.2
0.1
0.0
Absorbance 340nm
0
60
120
180
Time (Second)
Buffer NADP+ Riboflavin
NfrA NADP+ Riboflavin
Buffer NAD+ Riboflavin
NfrA NAD+ Riboflavin

g)

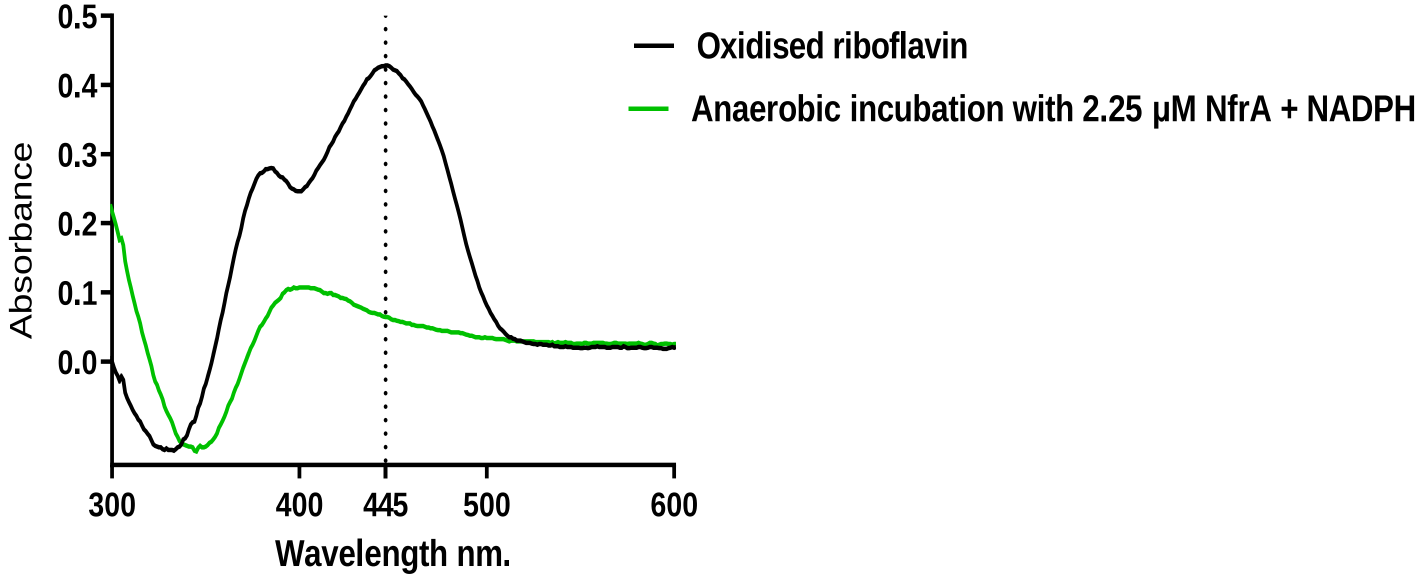

Fig. S3. Reductase activity of purified recombinant MdaB and NfrA. a) Oxidase activity of purified MdaB with NADPH and NADH (average of triplicates) and specific oxidase activity of MdaB calculated from initial rate (arrows indicate <0); **b)** Scans of all the quinones used in the study; **c)** Scans of all the quinone compounds tested; **d)** Traces of quinone compounds in the absence of MdaB (average of triplicates); **e)** Traces of FMN and FAD in the presence of of NfrA and NADPH (average of triplicates); **f)** Traces of riboflavin in the prescence of MdaB with NAD and NADP+ (average of triplicates)**g)** Spectrum scan performed pre- and post- assay to confirm quantitative reduction of riboflavin

Figure. S4:

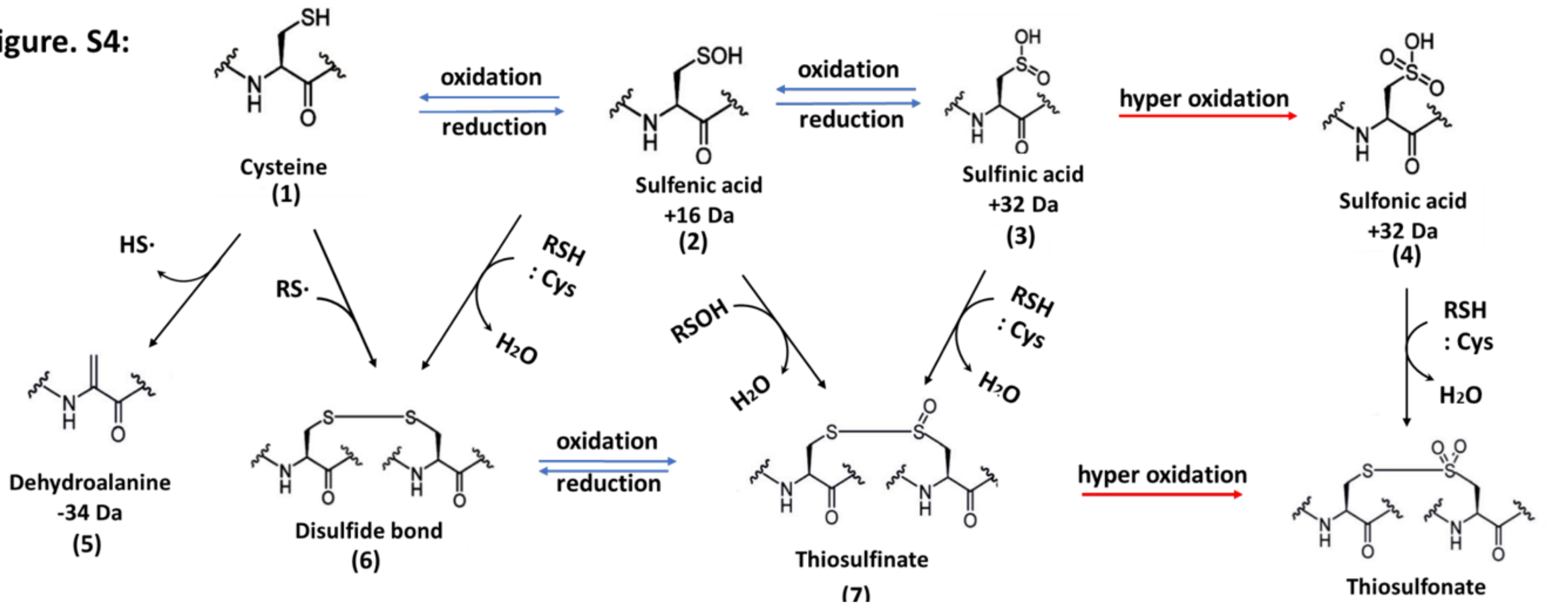

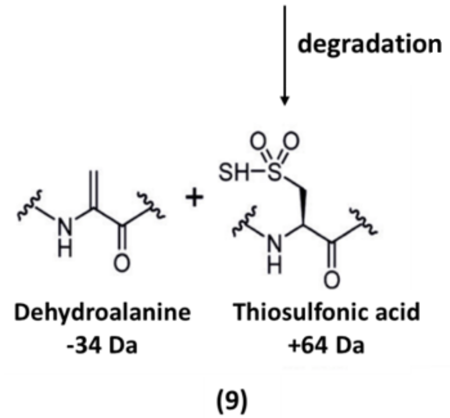

Figure S4. RrpA and RrpB hypothetical thiol based redox-switch mechanism.

Cys **(1)** thiol group can be reduced (desulfurization) by oxidants such as H2O2 to dehydroalanine, and can also be oxidized by H2O2 or other available ROS to sulfenic acid (intermediate state) **(2)**. sulfenic acid is generally unstable and is further oxidized to a stable sulfinic acid (-SO2H) **(3)** or forms a disulfide bond **(6)** with a nearby thiol group. Disulfide is the main oxidation product of Cys and its formation is necessary to change protein structures by oxidation. Sulfinic acid can be further oxidized to sulfonic acid (-SO3H) **(4)**. The sulfur atom of sulfenic acid can act as an electrophile as well as a nucleophile, this characteristic is key to the formation of disulfide bond and Thiosulfinate **(7)**. Similarly, Cys sulfinic acid can react with sulfenic acid to produce Cys thiosulfonate **(8)**. The same modifications can be obtained through oxidation of disulfides by oxidants such as hydrogen peroxide or singlet oxygen. Thiosulfonate can undergo degradation through oxidation to produce Thiosulfonic acid and Dha **(9)**. Figure adopted from Hee-Jung Kim *et al* 2015 [1].

Table. S4:

a) List of primers used for cloning

Gene Name **Primers**

rrpA	F 5'- ctttaagaaggagatatacatatgACTAAAGAGAATTCTCCGTGC-3'<br>R 5'- agtggtggtggtggtggtgctcgagATTCAAGCATTTTTTCCCTTTTTTG-3'
rrpB	F 5'- ctttaagaaggagatatacatatgAAAAAATATCATTCTCTTTTCC-3'<br>R 5'- agtggtggtggtggtggtgctcgagAACGATATTTTTATAGCTATTTCC-3'
*rrpA*cys8	F 5'- ctttaagaaggagatatacatatgACTAAAGAGAATTCTCCGTCCAAT-3'<br>R 5'- agtggtggtggtggtggtgctcgagATTCAAGCATTTTTTCCCTTTTTTG-3'
*rrpA*cys13	F 5'- ctttaagaaggagatatacatatgACTAAAGAGAATTCTCCGTGCAATTTCGAAGAATCTGG-3'<br>R 5'- agtggtggtggtggtggtgctcgagtcgagATTCAAGGATTTTTTCCCTTTTTTG-3'
*rrpA*cys33	F_1 5'- ctttaagaaggagatatacatatgACTAAAGAGAATTCTCCGTG-3'<br>R_1 5'- acgcacgattTCATAGCGAAATAAGGAGTATAAAATAC-3'<br>F_2 5'- ttcgctatgaAATCGTGCGTTATAATGAAC-3'<br>R_2 5'- agtggtggtggtggtggtgctcgagATTCAAGCATTTTTTCCCTTTTTTG-3'
*rrpA*cys113	F 5'- ctttaagaaggagatatacatatgACTAAAGAGAATTCTCCGTG-3'<br>R 5'- agtggtggtggtggtggtgctcgagATTCAAGGATTTTTTCCCTTTTTTG-3'
*rrpB*cys8	F 5'- ctttaagaaggagatatacatatgAAAAAATATCATTCTCTTTCTCC-3'<br>R 5'- agtggtggtggtggtggtgctcgagAACGATATTTTTATAGCTATTTCC-3'
mdaB	F 5'- ctttaagaaggagatatacatatgAAAAATATACTCTTGCTTAATGG-3'<br>R 5'- agtggtggtggtggtggtgctcgagAAAAATTTTTTTCAAGTGTAGTTC-3'
nfrA	F 5'- ctttaagaaggagatatacatatgAAAATAGCAATTTTATGTGCAAG-3'<br>R 5'-agtggtggtggtggtggtgctcgagTAGGCTTATTACCCCAATG-3'

F – Forward primer
R – Reverse primer
1- Primer used to generate fragment 1 (includes overlap to fragment2)
2- Primer used to generate fragment 2 (includes overlap to fragment1)
Lowercase = Overlapping sequence

b) List of primers and probes used for RT-qPCR

Gene name	Probe	Primer
gyrA	5' -(6-FAM) CCTTGCTTGAAAATTT-(MGB)	F 5'-AGTAATACGTGGCACATCAAATTTACTTCTAAT-3'<br>R 5'-GCAGAATTAATGAAAGAAATTGCAAGACTTG-3'
rrpA	5' -(6-FAM) AATACGCTCAAATTCC-(MGB)	F 5' -CAATACCTTAAGAGAACTAGAAAACGATGGT-3'<br>R 5' -ACTTTGTCCACGTTTGGATAAGCTA-3'
rrpB	5' -(6-FAM) CCTTGCAACAAATCTC-(MGB)	F 5' -CTATTGAAACCACGCTTAATTTGATAGGAA-3'<br>R 5' -AAATGAAATGCTTTTTCTTAACTCTCCAAATCTT-3'
mdaB	5' -(6-FAM) AAGCACTCCAGCACCC-(MGB)	F 5' -TTCCGTAGTTTTTACTAGGATTTTCATGAGTT-3'<br>R 5' -GGGAGCCTTGGATAGTTAAAAAATATATCG-3'
nfrA	5' -(6-FAM) CCGCACTTGCTTTAGC-(MGB)	F 5'-ACCTTTTTCATTGCGTAAAAATTCCAATACT-3'<br>R 5'-CAAGACTTATGGATATGCCTGATTTTCCT-3'
capA	5' -(6-FAM) ACCTTCGCCAGATAAA-(MGB)	F 5'-CGGCCTCTTTAAGTGGAGAAATGAT-3'<br>R 5'-CCAACACCTCTTCCACCTTCTAC-3'
rsmA	5' -(6-FAM) AACTCATCACCGCTGACAT-(MGB)	F 5'-TTCGCAAAGTTCATCAAA-3'<br>R 5'-CGCTAAAGAAGGAAATAGTG-3'

6-FAM - 6-carboxyfluorescein
MGB – Minor groove binder
F – Forward primer
R – Reverse primer

c) List of primers used for DNAse I footprinting and probes used for EMSA

Name	Primer/probe
**DNAse I footprinting**	
cj1546Ffam	5’-(6FAM)-AGCATGATTATGCAATGTCAAATTAAG-3’
cj1546Rhex	5’-(HEX)- TAATGCTAGAGTATAGTTAAATCCACAT-3
cj1556Ffam	5’-(6FAM)- TTGCGTACAAAACATACTGGCT-3
cj1556Rhex	5’-(6FAM)- AGTCCCTTGCAACAAATCTCGAATAA-3’
**EMSA**	
rrpAprobe	5’-/5IRD800/GCATAATATCATTTTCAATACAGAAAATAAAGTAATATACTATACTAAAGGAATGTTAAATGA-3’
mdaBprobe	5’-/5IRD800/AAATTTAAAATACTATGTTAATATTGAATCGTATTATAGTAAAAGTT-3’
rrpBprobe	5’-/5IRD800/TTAAAAAATATGTTATAATTTTAAAAAATAAGGATTTATAATGAAAAAATAT-3’
nfrAprobe	5’-/5IRD800/TAGATCTTTAATATTTATATAAATGATGATTTTTAGTTCATCTATAATAAAATATTATATCA-3’

6-FAM - 6-carboxyfluorescein
HEX - Hexachloro-fluorescein
IRD800 – Fluorophore dye
F – Forward primer
R – Reverse primer

Reference:

1. Kim, H.J., et al., *ROSics: chemistry and proteomics of cysteine modifications in redox biology.* Mass spectrometry reviews, 2015. **34**(2): p. 184-208.
